# Nagashima-Type Palmoplantar Keratosis: Clinical Characteristics, Genetic Characterization, and Clinical Management

**DOI:** 10.1155/2021/8841994

**Published:** 2021-01-27

**Authors:** Chao Huang, Yali Yang, Xingyu Huang, Zongke Zhou

**Affiliations:** ^1^Department of Orthopaedics, West China Hospital of Sichuan University, No. 37 Guoxue Alley, Wuhou District, Chengdu, 610041 Sichuan, China; ^2^Department of Dermatology, Shanghai Ninth Hospital Affiliated to Shanghai Jiao Tong University, School of Medicine, Shanghai 200011, China; ^3^Department of Dermatology, The First Medical Center of Chinese PLA General Hospital, 28 Fuxing Road, Haidian District, Beijing 100039, China

## Abstract

Nagashima-type palmoplantar keratosis (NPPK) is the most prevalent palmoplantar keratoderma (PPK) in East Asia. Homozygous or compound heterozygous loss-of-function mutations in serpin peptidase inhibitor, clade B (ovalbumin), and member 70 (SERPINB7), which encodes members of the serine protease inhibitor superfamily, have been identified as the cause of NPPK. Clinical manifestations of NPPK include well-demarcated erythema, mild to moderate hyperkeratosis on the whole palm, and sole with transgrediens, extending to the dorsal surfaces of the hands and feet, inner wrists, ankles, and the Achilles tendon areas. In this study, we perform a review of relevant clinical cases aimed at elucidating the clinical characteristics, genetic characterization, differential diagnoses, and clinical management of NPPK. A better understanding of the clinical characteristics and pathogenic gene characterization of NPPK will enhance the diagnosis of NPPK, identify related diseases, and inform on the precise therapy and prognosis. Moreover, it will promote the awareness of NPPK in non-Asian regions.

## 1. Introduction

Nagashima-type palmoplantar keratosis (NPPK, Online Mendelian Inheritance in Man no. 615598) was initially described in Japan by Masaji Nagashima in 1977. It is characterized by transgressive and nonprogressive keratosis with an autosomal recessive trait [1] and was once described as a mild form of mal de Meleda (MIM 248300). In 2008, Kabashima et al. introduced NPPK to international societies with a detailed description of the disease phenotype as a novel entity of PPK [2]. Homozygous or compound heterozygous loss-of-function mutations in serpin peptidase inhibitor, clade B (ovalbumin), member 70 (SERPINB7), have been identified as the cause of NPPK. To elucidate on NPPK, we performed an online search of the Embase, PubMed, Web of Science, and Wan-fang databases to retrieve related case reports or series of case reports regarding NPPK. This study presents the first comprehensive literature review of NPPK and provides a comprehensive review of the clinical characteristics, genetic characterizations, and therapeutic options for NPPK. Therefore, we believe that this review will provide an evidence-based reference for future clinical treatments and basic research for NPPK.

## 2. Clinical Features

**Figure 1:**
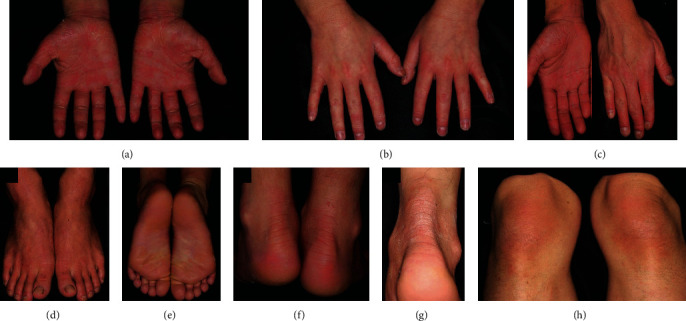
(a–g) Patients with NPPK showing diffuse erythema and hyperkeratosis on the entire palms and soles, extending to the dorsal surfaces of the hands and feet, ankles, and the Achilles tendon [7]. (h) Scaly erythema on the knee representing NPPK [7]. (All the images were obtained from copyright license holders without any conflict of interest).

**Figure 2:**
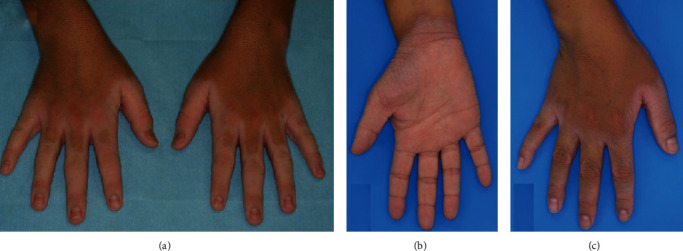
The affected skin is characterized by a white, spongy change within 10 minutes of contact with water [8, 9]. (All the images were obtained from copyright license holders without any conflict of interest).

NPPK is characterized by “transgrediens,” which refers to hyperkeratosis that extends beyond the palmar margin of the palmoplantar skin, primarily involving the palms, soles, dorsal surfaces of the hands and feet, inner wrist, ankle joint, and the Achilles tendon area [1, 3]. Notably, the elbows and knees are frequently affected. Moreover, a single case involving lesions on the extremities and lumbar area [4], ears [5], and nail [6] has been reported as atypical manifestations of NPPK (Figure 1). Reports also indicate high frequencies of hyperhidrosis on palms and soles, with tinea pedis as well as odor complications [2]. In addition, NPPK is characterized by a white, spongy change in the affected areas within 10 minutes of contact with water (Figure 2). Of note, NPPK is most prevalent among infants and young children, while its manifestations are less severe and nonprogressive after puberty. Clinical observations revealed no differences between males and females, and no change between seasons (Supplement, Table 1).

## 3. Biological Background

Studies on the etiology of NPPK have taken more than 30 years. Its first English literature report in 2008 elicited increased research focus and rapid progress on this subject [2]. Currently, NPPK has been reported in China [10], South Korea [11], and Finland [12] with many studies focusing on its pathogenic genes. Through whole-exome sequencing of 3 unrelated Japanese patients with NPPK in 2013, Kubo et al. discovered SERPINB7 as a pathogenic gene of NPPK that encodes a member of the serine protease inhibitor superfamily [1]. All the 3 patients were found to have a nonsense mutation, c. 796C>T, with homozygote or compound heterozygote in SERPINB7, which was in tandem with other findings. From the Human Genetic Variation Database and the 1,000 Genomes database, c.796C>T is prevalent in normal Japanese and Chinese populations, with frequencies of 1.12% and 1.52%, respectively [13]. Therefore, screening for c. 796C>T mutation has been recommended as a priority in patients suspected of having NPPK [10].

So far, more than 100 unrelated, molecularly diagnosed cases of NPPK associated with 15 distinct pathogenic SERPINB7 mutations in the homozygous or compound heterozygous state have been reported (Figure 2). This includes the most popular founder mutation (c.796C>T) and other potentially frequent mutations, including c.218_219delAGinsTAAACTTTACCT (c.218_219del2ins12), c.336+2T>G, c.455-1G>A, c.455G>T, c.522-523insT, c.522dupT, c.650_653delCTGT, c.830C>T, c.122_127delTGGTCC, c.635delG, c.382C>T, c.271delC, c.1136G>A, and c.521_522insT [1, 3, 9, 10, 12, 14–18], together with another putative causative mutation c.309delT in databases (Supplement, Table 2) [19].

**Figure 3:**
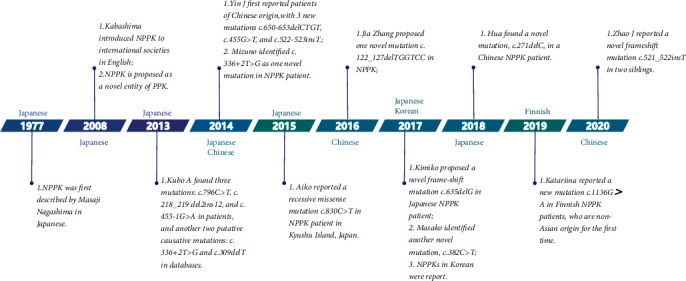
A timeline for the research progress of NPPK and the first report of each mutation of SERPINB7 gene.

The establishment of the etiology of NPPK has taken a long time (Figure 3). In 2013, Kubo et al. sequenced the genome of 13 patients diagnosed with NPPK and found 3 mutations of SERPINB7, including c.796C>T, c.218_219del2ins12, and c.455-1G>A [1]. These results confirm that c.796C>T and c.218_219 del2ins12 are the major mutations for NPPK in the Japanese population. After evaluating the variant database of the cohort of 1,092 individuals in the 1,000 Genomes Project [20], they found another 2 putative causative mutations: c.336+2T>G (an SNP of rs201433665) in Asian and c.309delT in non-Asian populations within databases, thereby predicting a premature stop codon (p. Phe103Leufs∗33). Therefore, they postulated that the c.796C>T mutation is the main mutation associated with NPPK in Asian populations. Moreover, the prevalence rate of NPPK is estimated at 1.2/10,000 in the Japanese populations and 3.1/10,000 in the Chinese populations, when compared to ~0.5/100,000,000 in the non-Asian populations [1]. All early reported NPPK cases were of Japanese origin; however, Yin et al. first reported patients of Chinese origin in 2014 [10]. Specifically, they reported 7 unrelated Chinese NPPK patients and discovered 3 new mutations underlying SERPINB7, including c.650-653delCTGT (p. S217Lfs∗7, frameshift mutation), c.455G>T (p.G152V, point mutation), and c.522-523insT (p. V175Cfs∗46, frameshift mutation). In the same year, Mizuno et al. analyzed samples from 10 Japanese families with NPPK and identified c.336+2T>G mutation in SERPINB7 as a novel mutation causing NPPK [3]. In January 2016, Aiko *et al.* reported a recessive missense mutation of SERPINB7, c.830C>T (p.P277L), in NPPK patients which causes the mislocalization of SERPINB7 to intracellular aggregates. The mutated protein aggregates and is mislocalized within corneocytes [14]. Later, in November 2016, Jia Zhang reported 12 unrelated Chinese NPPK patients, where another new mutation, i.e., c.122_127delTGGTCC (p. Leu41fs, in-frame deletion mutation), was found. This mutation shortens the protein and exerts pathogenic effects resulting in an NPPK phenotype [15]. In July 2017, Nakajima et al. found a novel mutation, c.635delG (p. K213Sfs∗12, frame-shift mutation), in a Japanese NPPK patient [9], while Katsuno et al. identified another novel mutation, c.382C>T (p.R128∗, nonsense mutation), in SERPINB7 [16]. In 2018, Hua *et al.* found a novel mutation, c.271delC (p. His91Thrfs∗9, frameshift mutation), in a Chinese NPPK patient, which formed a premature stop codon that truncated a 98-amino acid protein thereby eliminating a critical reactive site loop (P17–P50, amino acid residues: 331–352) [17]. In 2019, Hannula-Jouppi et al. reported another new mutation, c.1136G>A (p. Cys379Tyr), in Finnish NPPK patients of non-Asian origin [12]. According to the Genome Aggregation Database (GnomAD), the heterozygous carrier frequency was 5- to 20-fold higher in the Finnish population (0.006397) compared to non-Finns (0.00032-0.0014), indicating that the heterozygous carrier constituted a plausible Finnish NPPK founder mutation [12]. In addition, they recommended the assessment of SERPINB7 mutations in non-Asian individuals with an NPPK-phenotype [12]. A recent study by Zhao et al. reported a compound heterozygous mutation, c.796C>T/c.521_522insT, in 2 siblings [18]. The c.521_522insT is a novel frameshift mutation at exon 6 that changes the amino acid sequence to start with Val 175 and later terminating the polypeptide at 46 amino acids [18].

## 4. Pathogenesis

The etiology of NPPK has been established. However, its pathogenesis has not been clearly elucidated. The SERPINB7 gene, located in 18q21.3, encodes the serine protease inhibitor B subtype 7, which inhibits serine protease and protects cells from protease-mediated damage [1, 21]. The active site ring (reactive site loop, RSL) of SERPINB7 is located at 331-352 amino acid, where the basic condition for maintaining its activity is to preserve the integrity of this functional region [22, 23]. All known mutations trigger truncation of the active site ring, resulting in the noninhibition of SERPINB7 protease activity in the granular layer and stratum corneum in the epidermis of NPPK patients. Additionally, it causes the downregulation of SERPINB7 in the epidermis, which cannot effectively inhibit serine protease activity, resulting in protein degradation of keratinocytes, destruction of cuticle barrier functions, and facilitating water permeation into the stratum corneum. Consequently, this leads to a series of clinical manifestations including hyperkeratosis, skin swelling, and whitening [1].

SERPINB7 is distributed in the epidermis throughout the body, particularly in the stratum granulosum and upper part of the stratum corneum, whereas NPPK is limited to the palms, soles, knees, and elbows. The pathomechanism of NPPK lesions is usually restricted to specific areas that remain unknown. Frequent involvement of the knees and elbows in NPPK imply that chronic exposure to mechanical stress might be involved in the development of NPPK lesions, by precisely inhibiting mechanical stress-induced proteases and protecting keratinocytes or corneocytes from protease-mediated cellular damage [1, 19]. However, there is a need to determine why NPPK-related skin lesions are limited to the palmoplantar skin and the role of SERPINB7 in the pathogenesis of hyperkeratosis and erythema in the palmoplantar skin.

**Figure 4:**
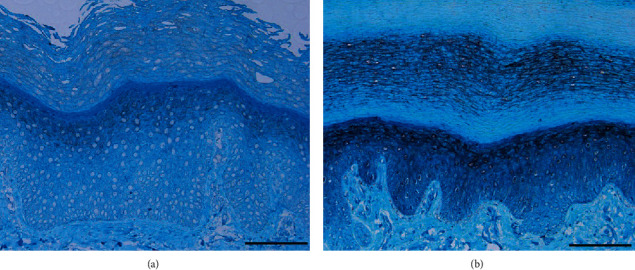
(a, b) Immunohistochemistry using a polyclonal antibody raised against a peptide corresponding to amino acids 203–334 of human SERPINB7 showing a lack of SERPINB7 immunostaining at the stratum corneum in the proband (a), but not in the normal control (b) [16]. (All the images were obtained from copyright license holders without any conflict of interest).

Given that T cells have been shown to be infiltrated in the affected lesions, it is important to focus on the possible role of T cell-mediated inflammation in the pathogenesis of this superficial genetic disease. In 2009, Sakabe et al. performed immunohistochemical staining on a sample from the skin of NPPK patients and found that CD4^+^ T lymphocytes had infiltrated the diseased tissue [24]. Subsequently, they topically applied an immunosuppressive therapy and obtained a satisfactory outcome, suggesting that NPPK can be induced or exacerbated by T cells infiltrating into the skin [24]. Elsewhere, another set of immunohistochemical findings indicated that the expression of SERPINB7 was significantly low in NPPK lesions (Figures 4(a) and 4(b)) [16], while Keratin (KRT) 1 and KRT14 were strongly expressed in the cytoplasm of keratinocytes in the epidermis of NPPK skin lesion. This implies that the loss of SERPINB7 can be compensated by upregulating KRT1 and KRT14 [25]. In addition, the expression of proteins that influence epidermal differentiation, such as loricrin and filaggrin, was not affected, implying that there was no apparent keratinocyte differentiation defect in the skin of the NPPK-affected area.

Since its pathogenesis remains elusive, it is crucial to clarify the mode of its inheritance. NPPK is an autosomal recessive disease, mostly originating from unrelated family mating. However, in some cases, the patients show pseudodominance, an autosomal recessive condition which occurs like the autosomal-dominant inheritance model amongst individuals in 2 or more generations of a family [3, 4, 26]. Further, SERPINB7 null mutations are expected to be prevalent in the Chinese and Japanese populations, with the combined SERPINB7 null allele frequency in these populations being 0.015 and 0.011, respectively, which also promotes pseudodominance in families with NPPK [1, 4]. Therefore, a more accurate genetic testing and counseling of this disease is essential.

## 5. Clinicopathologic Characterization

**Figure 5:**
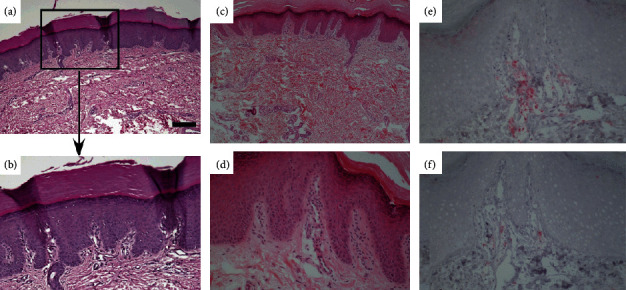
Histological findings from the affected lesion. (a, b) A biopsy specimen showing orthokeratotic hyperkeratosis, mild hypergranulosis, and acanthosis, with a mild sparse infiltrate of lymphocytes in the upper dermis noted in a high-power view [32]. (c–f) The affected skin shows orthokeratotic hyperkeratosis with a mild to moderate infiltrate of mononuclear cells in the dermal papilla, and immunohistochemistry with anti-CD4 (e) and CD8 (f) monoclonal antibodies showing the majority of lymphocytes being CD4^+^T cells [24]. (All the images were obtained from copyright license holders without any conflict of interest).

Histopathology of the diseased skin of NPPK is nonspecific and exhibits similar manifestations with other PPK. It is primarily characterized by hyperkeratosis, hypergranulosis, and incomplete keratosis of the epidermis, hypertrophy of the granular layer and spinous layer, infiltration of a small number of lymphocytes in the superficial layer of the dermis in a high-power view, and a marked increase in the glandular portion of the eccrine sweat glands at the dermis–hypodermis interface, without epidermolysis, viral inclusion, granular degeneration, or epithelial cell abnormalities (Figure 5) [2, 11, 16]. In a few patients with NPPK, immunohistochemistry showed that a few CD3^+^ T cells were in the dermis with hyperkeratosis. Analysis of the infiltrating cells showed that they primarily included CD3^+^CD4^+^ T and a few CD3^+^CD8^+^ T cells, without CD1a^+^ and Langerin^+^ epidermal Langerhans cells, or exocytosis [27]. Despite reports on mild T cell infiltration in the affected skin area, the pathophysiology of the skin redness and hyperkeratosis has not been characterized [24]. Kogame *et al.* reported that the hyperkeratosis and thickening of PPK might reflect the severity of inflammation and oxidative stress, which deteriorates the local microenvironment [28–30]. Furthermore, under an electron microscope, NPPK shows the lysis of keratinocytes between keratin desmosomes and excessive exocytosis of keratinocytes, which might trigger excessive skin adhesion. At the same time, a large number of lipid inclusion bodies exist in the granular layer [31].

## 6. Differential Diagnosis

The clinical features of diffuse PPK and recessive inheritance are not unique to NPPK. Thus, it is necessary to distinguish it from other diseases during clinical diagnosis and treatment (Table 1). Gene sequencing plays a significant role in the diagnosis. Moreover, NPPKs can be overlooked if they are accompanied by other dermatoses, such as atopic dermatitis and melanoma. As such, dermatologists should draw attention to characteristic lesions of NPPK in such cases [7].

## 7. Management and Prognosis

Although the NPPK phenotype is milder when compared to that of other hereditary palmoplantar hyperkeratosis and exhibits a nonprogressive disease progression, an effective treatment is still needed when the high allele gene frequency of founder mutation (c. 796C>T) in Chinese and Japanese populations, as well as discomfort and psychological burden of this disease, is considered. A standard therapeutic option for NPPK is currently unavailable. Therefore, the current management approaches for this disease is mainly aimed at reducing hyperkeratosis using topical vitamin D3 and/or topical keratolytic agents, including 5%, 10% salicylic acid, 10% urea, and adapalene [2, 4, 47]. Hyperhidrosis and odor are the frequent complaints from NPPK patients [2], and topical 10% aluminum potassium sulfate lotion and 2.5% benzoyl peroxide gel are applied to counter hyperhidrosis and odor, respectively [16]. A combination of NPPK with fungal infection and atopic dermatitis necessitates the application of antifungal drugs and steroid therapy, respectively. However, these treatments can only temporarily relieve symptoms, which might recur after stopping drug intake, thus indicating unsatisfactory outcomes.

In 2009, Sakabe et al. performed immunohistochemical staining on a simple affected skin of NPPK patients and found that it was infiltrated by CD4^+^ T lymphocytes [24]. They applied 0.1% tacrolimus ointment and 0.05% betamethasone butyrate propionate ointment to the left and right hand, respectively. The efficacy of immunosuppressive agents and glucocorticoids was then compared. They found that erythema and hyperkeratosis of both hands were improved with both treatments. The improvement was, however, more significant on the left compared to the right hand, implying that topical immunosuppressive therapy exhibits a certain therapeutic potential for NPPK [24].

Approximately one-third of alleles that cause genetic diseases carry premature termination codon (PTC), which trigger the production of truncated proteins [48]. Moreover, about 10% of genetic diseases are caused by nonsense mutations where a stop codon is introduced to prematurely terminate the synthesis of full-length protein [49]. Gentamicin, specifically gentamicin B1, induces the read-through of nonsense mutations. Cells in the affected lesion can bypass the termination codon, insert random amino acids, and express full-length proteins by interfering with the proofreading ability of mRNA. Gentamicin is used to treat hereditary diseases caused by premature termination codons, such as cystic fibrosis, Duchenne muscular dystrophy, and Hailey-Hailey disease [48, 50]. From literature, the nonsense mutation, c. 796C>T (p. Arg266Ter), in the last exon of SERPINB7, is the most common among the pathogenic genes of NPPK. Based on the above findings, Ohguchi et al. studied the effects of gentamicin in the treatment of NPPK in 2018 [13]. They first transfected 293 cells with SERPINB7 cDNA with c. 796C>T (p. Arg266Ter) mutation. They found that gentamicin could induce the reading and expression of the full-length SERPINB7 protein in the transfected 293 cells and immortalized primary keratinocytes of an NPPK patient who was homozygous for the c.796C>T mutation. Subsequently, in 5 NPPK patients with c.796C>T (p. Arg266Ter) mutation, 0.1% gentamicin ointment (qd, 0.5 g) was topically applied for 4 weeks, which effectively reduced the degree of hyperkeratosis of skin lesions in NPPK patients, but could not effectively improve erythema. This was attributed to [13] (i) more SERPINB7 synthesis was required to completely cure the disease or (ii) gentamicin-induced SERPINB7 might not be fully functional, because aminoglycosides induce ribosomes to incorporate random amino acids through near-source aminoacyl transfer RNAs and read through PTCs. At the same time, local low-dose application of gentamicin exhibited a significant effect and prevented the potential ototoxicity and nephrotoxicity of aminoglycoside antibiotics [13]. Nonetheless, the long-term safety and curative effect of this therapy should be evaluated by follow-up studies. Besides, a large case-control study is necessary in the future [17].

## 8. Conclusion and Future Perspective

Due to advances in genetic testing technologies in NPPK diagnosis and treatment as well as the accumulating number of related case reports, NPPK has gradually been recognized by scholars across the globe. However, an internationally recognized diagnostic criteria for NPPK are currently lacking. Notably, the possibility of NPPK cannot be ruled out in patients with clinically sporadic, nondestructive, diffuse palmoplantar keratosis. Genetic testing technologies can be used to confirm the diagnosis of NPPK by integrating medical history, clinical manifestations, and laboratory examination of the patients for differential diagnosis. During diagnosis by genetic testing, priority is given to the detection of the founder mutation (c.796C>T), which exhibits the highest frequency among SERPINB7 mutations. Moreover, considering the high allele frequency of the founder mutation in normal people, it is essential to conduct genetic counseling for NPPK patients or NPPK carriers and their partners. Despite the effectiveness of local immunosuppressants and aminoglycoside antibiotic read-through therapies, their long-term effects have not been determined. More studies should be aimed at elucidating the pathogenesis of NPPK and identifying a precise therapy with stable curative effects.

## Figures and Tables

**Table 1:** The major clinical differentiating characteristics for distinguishing NPPK from other diseases with similar characteristics.

	NPPK [19]	MDM PPK [2, 33]	Greither PPK [34]	PPK-GN [35]	Bothnian PPK [36, 37]	Acral keratoderma [2]	Symmetrical lividities of the soles [38]	PSEK [39]
Hyperkeratosis	Mild and nonprogressive	Severe	Thick	Thick	Mild to thick	Thick	NM	+
Hyperhidrosis	+	+	+	NM	+	NM	+	NM
Flexion contractures	–	+	–	–	–	–	NM	NM
Constricting bands surrounding the digits	–	+	+	+ (occasionally)	–	+	NM	NM
Spontaneous amputation	–	+ (occasionally) [40, 41]	+	NM	–	+	NM	NM
Genetic model	AR	AR	AD	AR	AD	AR	NM	AD
Pathogenic gene	SERPINB7	SLURP1 [42]	KRT1 [34]	Unknown	AQP5 [36, 37]	TCF4 [43, 44]	NM	NM
Prevalence rate	Japan: 1.2/10,000; China: 3.1/10,000 [1]	Island of Meleda: common; General populations: 1/100,000	Rare	Rare	Rare	Rare	Rare	Rare
Whitish spongy change in affected areas upon exposure to water	+	–	–	–	+	–	NM	NM
Transgrediens	+	+	+	+	+	+	NM	NM
Progressive clinical presentation	- (after puberty)	+ (throughout the lives)	NM	NM	NM	NM	NM	NM
Special clinical features in addition to mentioned above		Perioral erythema, brachydactyly, nail abnormalities, and lichenoid plaques [41]; consanguineous marriage [45, 46]		The dorsal aspects of the finger joints are covered by hyperkeratotic plaques [35]	More hyperhidrotic, mild nail changes, and more rapid and obvious whitish spongy change and swelling upon exposure to water	Striate hyperkeratinosis of the palms and soles, and linear hyperkeratotic lesions over the Achilles tendon areas, ankles, elbows, and knees [2]	Symmetrical, bluish-red plaques on the soles of the feet, and rare involvement of palm [38]	Non-migratory erythematous and hyperkeratotic plaques that are distributed symmetrically over the body

NPPK: Nagashima-type palmoplantar keratosis; MDM: mal de Meleda; PPK-GN: Gamborg-Nielsen type PPK; PSEK: progressive symmetrical erythrokeratoderma; NM: not mentioned; +: present; −: not present; AR: autosomal recessive inheritance; AD: Autosomal dominant inheritance.

## Data Availability

The table data used to support the findings of this study are included within the supplementary information files.

## Supplementary Materials

Supplementary Materials Supplement, Table 1: Clinical phenotypes in individuals with NPPK. Supplement, Table 2: SERPINB7 mutations in individuals with NPPK.
