# Supplementary material for: Nagashima-Type Palmoplantar Keratosis: Clinical Characteristics, Genetic Characterization, and Clinical Management
Source: Biomed Res Int. 2021 Jan 27;2021:8841994. doi: 10.1155/2021/8841994 (PMC7861918; doi:10.1155/2021/8841994)
Supplement: Supplementary Materials — Supplement, Table 1: Clinical phenotypes in individuals with NPPK. Supplement, Table 2: SERPINB7 mutations in individuals with NPPK. [file 8841994.f1.zip › Supplement-Table 1 (1).docx]

**Supplement, Table 1:** Clinical phenotypes in individuals with NPPK.

| **No** | **Source** | **Year** | **Country** | **Sex/Age** | **Onset** | **Clinical features of PPK** | **Involved areas** | **Other Involved areas** | **Hyperhidrosis** | **Aquagenic whitening** | **Clinical features in addition to PPK** | **Family history** | **Treatment** | **Outcome** |
| --- | --- | --- | --- | --- | --- | --- | --- | --- | --- | --- | --- | --- | --- | --- |
| 1 | Zhao et al.^[1]^ | 2020 | China | F/22mo | 1y | Nonpruritic diffuse erythema with thin desquamation | Palms and soles | NM | NM | NM | NM | Her sister | NM | NM |
| 2 | Zhao et al.^[1]^ | 2020 | China | F | NM | Diffuse erythema and hyperkeratosis with transgrediens | Hands and feet | NM | NM | NM | NM | P1' sister | NM | NM |
| 3 | Zhao et al.^[1]^ | 2020 | China | M/3y | 6mo | Reddish and hyperkeratotic maceration lesions | Dorsal surfaces of the hands and feet | NM | NM | NM | Atopic dermatitis, urticaria, severe pruritus and odor. |  |  |  |
| 4 | Zhao et al.^[1]^ | 2020 | China | F/5y | Birth | Red patches | Palms and soles | NM | NM | NM | Atopic dermatitis, scratchy eruptions on the face, which spread to the trunk and extremities as she aged, particularly affecting the neck, cubital fossa, and popliteal areas. |  |  |  |
| 5 | Zhao et al.^[1]^ | 2020 | China | F | Birth | Nonpruritic diffuse erythema with thin desquamation | Palms and soles | NM | NM | NM | Atopic dermatitis, erythema and ﬁne scale behind the ears, elbows, and knees, accompanied by moderate pruritus. |  |  |  |
| 6 | Zhao et al.^[1]^ | 2020 | China | M | Birth | Nonpruritic diffuse erythema with thin desquamation | Palms and soles | NM | NM | NM | Atopic dermatitis, mildly pruritic erythema on his face |  |  |  |
| 7 | Zhao J^[2]^ | 2019 | China | M/4y,8mo | 2y | Erythema | Palms and soles | Wrist, ankles | + | + | Distinct odor |  | NM | NM |
| 8 | Zhao J^[2]^ | 2019 | China | M/17y | 4y | Reddish, keratosis | Palms and soles | Wrist, ankles, and Achilles tendon | + | + | Distinct odor |  | NM | NM |
| 9 | Sun et al.^[3]^ | 2019 | China | F/29y | 6mo | Diffuse erythema | Palms and soles | Wrist, ankles, and Achilles tendon | + | + | Mild odor |  | NM | NM |
| 10 | Katayama et al.^[4]^ | 2019 | Japan  (Chinese) | F/45 | 10y | Bilateral erythematous hyperkeratotic lesions, transgrediens | Palms and soles | NM | NM | + | Recurrent episodes of tinea pedis | Her siblings presented with similar hyperkeratotic lesions, whereas her parents were not affected. | The black macular lesion was excised with a 5-mm margin and the wound was reconstructed using a full-thickness skin graft from the right abdomen. |  |
| 11 | Hannula-Jouppi et al.^[5]^ | 2019 | Finland | M/27y | 2mo | Mild diffuse PPK with a well-demarcated erythema, transgradients | NM | Wrist and Achilles tendon area | + | + | Fungal infections | His mother and sister were variant heterozygous carriers. |  |  |
| 12 | Hannula-Jouppi et al.^[5]^ | 2019 | Finland | M/18y | Birth | Diffuse mild PPK, transgradients | NM | Wrist and Achilles tendon area | + | + | NM |  |  |  |
| 13 | Hannula-Jouppi et al.^[5]^ | 2019 | Finland | M/11y | 1.5y | Diffuse mild PPK, transgradients | NM | Wrist and Achilles tendon area | + | + | NM |  |  |  |
| 14 | Hannula-Jouppi et al.^[5]^ | 2019 | Finland | M/60y | Early childhood | Diffuse mild PPK, transgradients | NM | NM | + | NM | NM |  |  |  |
| 15 | Hannula-Jouppi et al.^[5]^ | 2019 | Finland | F/21y | Early childhood | Diffuse mild PPK, transgradients | NM | Wrist and Achilles tendon area | + | + | Fungal infections |  |  |  |
| 16 | Hannula-Jouppi et al.^[5]^ | 2019 | Finland | F/12y | Birth | Diffuse mild PPK, transgradients | NM | Wrist and Achilles tendon area | + | + | Fungal infections |  |  |  |
| 17 | Hannula-Jouppi et al.^[5]^ | 2019 | Finland | M/16y | 9y | Diffuse mild PPK, transgradients | NM | NM | + | + | Fungal infections |  |  |  |
| 18 | Korekawa et al.^[6]^ | 2019 | Japan | F/65y | NM | Hyperkeratosis | Palms and soles | NM | NM | NM | Malignant melanoma on her left fourth toe |  |  |  |
| 19 | Korekawa et al.^[6]^ | 2019 | Japan | F/85y | NM | Hyperkeratosis | Palms and soles | NM | NM | NM | Malignant melanoma on her right hand |  |  |  |
| 20 | Korekawa et al.^[6]^ | 2019 | Japan | M/55y | NM | Hyperkeratosis | Palms and soles | NM | NM | NM | Malignant melanoma on his right sole |  |  |  |
| 21 | Korekawa et al.^[6]^ | 2019 | Japan | M/38y | NM | Hyperkeratosis | Palms and soles | NM | NM | NM | Malignant melanoma on his right sole |  |  |  |
| 22 | Chassain et al.^[7]^ | 2019 | France  (Chinese P) | F/6y | Birth | Report confirmed NPPK | NM | NM | NM | NM | NM |  |  |  |
| 23 | Matsudate et al.^[8]^ | 2019 | Japan | M/9y | NM | Well-demarcated diffuse palmoplantar hyperkeratosis and mild erythema | Dorsum of the hands and feet | Inner wrists, knees, Achilles tendon area | NM | + | X-linked ichthyosis | His mother had a heterozygous mutation but no palmoplantar hyperkeratosis. |  |  |
| 24 | Liu C, Li CX^[9]^ | 2018 | China | M/23y | 2y | Erythema, keratosis macules | Palms and soles | Elbows, ankles, and Achilles tendon | + | + | NM |  | NM | NM |
| 25 | Hua et al.^[10]^ | 2018 | China | M/6y | Birth | Reddish, palmoplantar, hyperkeratotic lesions with erythematous border | Dorsa of the hands and feet. | NM | + | NM | Desquamation |  |  |  |
| 26 | Kogam et al.^[11]^ | 2018 | Japan | F/64y | Birth | Bilateral erythematous hyperkeratotic lesions | Palms and soles | NM | NM | NM | NM |  | Monthly topical injections of IFN-beta. |  |
| 27 | Ohguchi et al.^[12]^ | 2018 | Japan | M/28y | NM | Report confirmed NPPK | NM | NM | NM | NM | NM |  | Gentamicin (one hand) + Vaseline (one hand) | Improved |
| 28 | Ohguchi et al.^[12]^ | 2018 | Japan | M/29y | NM | Report confirmed NPPK | NM | NM | NM | NM | NM |  | Gentamicin (one hand) + Vaseline (one hand) | No improvement |
| 29 | Ohguchi et al.^[12]^ | 2018 | Japan | F/6y | NM | Report confirmed NPPK | NM | NM | NM | NM | NM |  | Gentamicin (one hand) + Vaseline (one hand) | No  improvement |
| 30 | Ohguchi et al.^[12]^ | 2018 | Japan | F/40y | NM | Report confirmed NPPK | NM | NM | NM | NM | NM |  | Gentamicin (one hand) + Vaseline (one hand) | No  improvement |
| 31 | Ohguchi et al.^[12]^ | 2018 | Japan | M/46y | NM | Report confirmed NPPK | NM | NM | NM | NM | NM |  | Gentamicin (one hand) + Vaseline (one hand) | Improved |
| 32 | Yamauchi et al.^[13]^ | 2018 | Japan | M/10y | Birth | Reddish and hyperkeratotic maceration | Palms and soles,  extending to the dorsal surfaces of the hands and feet | Inner wrists, ankles and Achilles tendon area | NM | NM | Atopic dermatitis, IgE(+) |  |  |  |
| 33 | Yamauchi et al.^[13]^ | 2018 | Japan | F/12y | Birth | Hyperkeratotic scaly erythema | Palms and soles | NM | NM | NM | Atopic dermatitis, scratchy eruptions on the face, trunk and extremities, IgE(+) |  | Topical steroid ointments | Poor efficacy |
| 34 | Yamauchi et al.^[13]^ | 2018 | Japan | M/20y | 1y | Reddish hyperkeratosis | Palms and soles | NM | NM | NM | Asthma, atopic dermatitis, IgE(+) |  | Topical steroid ointments | Poor efficacy |
| 35 | Yang et al.^[14]^ | 2017 | China | M/18y | 4y | Symmetrically distributed erythema | Palms and soles | Wrist, ankles, and Achilles tendon | + | NM | Distinct odor |  | NM | NM |
| 36 | Dai et al.^[15]^ | 2017 | China | M/17y | Infancy | Erythema, scaling | Palms and soles | Wrist, ankles, and Achilles tendon | + | + | Mild odor |  | NM | NM |
| 37 | Dai et al.^[15]^ | 2017 | China | M/26y | 2y | Reddish, keratosis, skin thickening and scaling | Palms and soles | NM | - | NM | Distinct odor, fungi (+) |  | NM | NM |
| 38 | Dai et al.^[15]^ | 2017 | China | F | NM | Suspected NPPK performance | NM | NM | NM | NM | NM | P37' mother | NM | NM |
| 39 | Dai et al.^[15]^ | 2017 | China | F | NM | Suspected NPPK performance | NM | NM | NM | NM | NM | P37' aunt | NM | NM |
| 40 | Dai et al.^[15]^ | 2017 | China | F | NM | Suspected NPPK performance | NM | NM | NM | NM | NM | P37' grandma | NM | NM |
| 41 | Qiu M, Zou XB^[16]^ | 2017 | China | F/28y | 7 mo | Scaly erythema | Palms and soles | Distal forearms, elbows, shins, knees, lower thighs and perianal area. | + | NM | Tinea pedis, intense pruritus; leukokeratosis and fissures were found on her tongue | Her mother | Betamethasone valerate 0.1% and gentamicin sulfate ointment | No improvement. |
| 42 | Tsutsumi et al.^[17]^ | 2017 | Japan | F/63y | Infant | Erythematous hyperkeratotic lesions | Palms and soles (the dorsal surfaces of the hands and feet) | Achilles tendon area | NM | + | Malignant melanoma on her left sole |  |  |  |
| 43 | Katsuno  et al.^[18]^ | 2017 | Japan | M/63y | 30y | Hyperkeratosis | Palmoplantar skin, dorsal skin of the hands and feet | Flexor aspects of the wrists and the Achilles tendon area | + | + | Odor on the soles |  |  |  |
| 44 | Hashimoto et al.^[19]^ | 2017 | Japan | NM | NM | Report confirmed NPPK | NM | NM | NM | NM | NM |  |  |  |
| 45 | Hashimoto et al.^[19]^ | 2017 | Japan | NM | NM | Report confirmed NPPK | NM | NM | NM | NM | NM |  |  |  |
| 46 | Hashimoto et al.^[19]^ | 2017 | Japan | NM | NM | Report confirmed NPPK | NM | NM | NM | NM | NM |  |  |  |
| 47 | Hashimoto et al.^[19]^ | 2017 | Japan | NM | NM | Report confirmed NPPK | NM | NM | NM | NM | NM |  |  |  |
| 48 | Hashimoto et al.^[19]^ | 2017 | Japan | NM | NM | Report confirmed NPPK | NM | NM | NM | NM | NM |  |  |  |
| 49 | Hashimoto et al.^[19]^ | 2017 | Japan | NM | NM | Report confirmed NPPK | NM | NM | NM | NM | NM |  |  |  |
| 50 | Hashimoto et al.^[19]^ | 2017 | Japan | NM | NM | Report confirmed NPPK | NM | NM | NM | NM | NM |  |  |  |
| 51 | Hashimoto et al.^[19]^ | 2017 | Japan | NM | NM | Report confirmed NPPK | NM | NM | NM | NM | NM |  |  |  |
| 52 | Hashimoto et al.^[19]^ | 2017 | Japan | NM | NM | Report confirmed NPPK | NM | NM | NM | NM | NM |  |  |  |
| 53 | Hashimoto et al.^[19]^ | 2017 | Japan | NM | NM | Report confirmed NPPK | NM | NM | NM | NM | NM |  |  |  |
| 54 | On et al.^[20]^ | 2017 | Korean | M | NM | Diffuse, erythematous palmoplantar hyperkeratosis | Dorsum of the hands/feet | Wrist, Achilles tendon | + | + | Recurrent tinea pedis |  |  |  |
| 55 | On et al.^[20]^ | 2017 | Korean | M | NM | Diffuse, erythematous palmoplantar hyperkeratosis | Dorsum of the hands/feet | Wrist, Achilles tendon | + | + | Recurrent tinea pedis |  |  |  |
| 56 | On et al.^[20]^ | 2017 | Korean | F | NM | Diffuse, erythematous palmoplantar hyperkeratosis | Dorsum of the hands/feet | Wrist, Achilles tendon | + | + | NM |  |  |  |
| 57 | Zhang et al.^[21]^ | 2017 | China | M/8mo | 2mo | Non-progressive, symmetrical, diffuse erythema and hyperkeratosis | Palms and soles | NM | NM | NM | NM |  |  |  |
| 58 | Zhang et al.^[21]^ | 2017 | China | F/2y | 1wk | Non-progressive, symmetrical, diffuse erythema and hyperkeratosis | Palms and soles | NM | NM | NM | NM |  |  |  |
| 59 | Zhang et al.^[21]^ | 2017 | China | F/19y | 3mo | Non-progressive, symmetrical, diffuse erythema and hyperkeratosis | Palms and soles | NM | NM | NM | NM |  |  |  |
| 60 | Zhang et al.^[21]^ | 2017 | China | M/4y | 6mo | Non-progressive, symmetrical, diffuse erythema and hyperkeratosis | Palms and soles | NM | NM | NM | NM |  |  |  |
| 61 | Zhang et al.^[21]^ | 2017 | China | M/16y | 3mo | Non-progressive, symmetrical, diffuse erythema and hyperkeratosis | Palms and soles | NM | NM | NM | NM |  |  |  |
| 62 | Zhang et al.^[21]^ | 2017 | China | F/26y | 2mo | Non-progressive, symmetrical, diffuse erythema and hyperkeratosis | Palms and soles | NM | NM | NM | NM |  |  |  |
| 63 | Zhang et al.^[21]^ | 2017 | China | F/17y | 2mo | Non-progressive, symmetrical, diffuse erythema and hyperkeratosis | Palms and soles | NM | NM | NM | NM |  |  |  |
| 64 | Zhang et al.^[21]^ | 2017 | China | F/24y | 5mo | Non-progressive, symmetrical, diffuse erythema and hyperkeratosis | Palms and soles | NM | NM | NM | NM |  |  |  |
| 65 | Zhang et al.^[21]^ | 2017 | China | F/36y | NM | Non-progressive, symmetrical, diffuse erythema and hyperkeratosis | Palms and soles | NM | NM | NM | NM |  |  |  |
| 66 | Zhang et al.^[21]^ | 2017 | China | M/22y | 3mo | Non-progressive, symmetrical, diffuse erythema and hyperkeratosis | Palms and soles | NM | NM | NM | NM |  |  |  |
| 67 | Zhang et al.^[21]^ | 2017 | China | F/51y | NM | Non-progressive, symmetrical, diffuse erythema and hyperkeratosis | Palms and soles | NM | NM | NM | NM |  |  |  |
| 68 | Zhang et al.^[21]^ | 2017 | China | F/2y | 2mo | Non-progressive, symmetrical, diffuse erythema and hyperkeratosis | Palms and soles | NM | NM | NM | NM |  |  |  |
| 69 | Li et al.^[22]^ | 2017 | China | M/45y | NM | Bilateral reddish, palmoplantar hyperkeratotic lesions (well-demarcated erythema and hyperkeratosis symmetrically) | Dorsa of the hands and feet | Wrists, ankles and Achilles tendon | NM | NM | NM |  |  |  |
| 70 | Nakajima et al.^[23]^ | 2017 | Japan | M/12y | 2y | Bilateral, reddish, palmoplantar, diffuse hyperkeratotic skin lesions with an erythematous border | Dorsum of the hands and feet | Flexor aspects of the wrists and ankles, the Achilles tendon area | - | + | NM |  |  |  |
| 71 | Duo et al.^[24]^ | 2016 | China | F/24y | birth | Erythema, scaling | Palms and soles | Wrist, ankles, and Achilles tendon | + | + | Fungal infection |  | 10% urea and 5% salicylic acid ointment for external use | Mild remission and relapse after withdrawal |
| 72 | Duo et al.^[24]^ | 2016 | China | F/25y | birth | Reddish, keratosis, skin thickening and scaling | Palms and soles | Wrist, ankles, and Achilles tendon，elbows and knees | + | + | Tinea pedis |  | 10% urea and 5% salicylic acid ointment for external use | Mild remission and relapse after withdrawal |
| 73 | Miyauchi et al.^[25]^ | 2016 | Japan | M/18y | Early childhood | Diffuse erythema with mild hyperkeratosis (bilateral, well-demarcated erythematous hyperkeratotic lesions) | Dorsal surfaces of the hands and feet | Inner wrists, ankles, the Achilles tendon area, Knees and elbows, extremities and lumbar | + | + | NM |  | 10% salicylic acid and vitamin D3 | Little improvement |
| 74 | Tokimasa et al.^[26]^ | 2015 | Japan | F/16y | 3mo | Diffuse erythematous hyperkeratosis | Palms and soles, dorsal aspect of the ﬁngers | Inner wrists and ankles, erythema on the elbows and knees | + | + | NM |  |  |  |
| 75 | Masashi et al.^[27]^ | 2014 | Japan | F/64y | Birth | Bilateral erythematous hyperkeratotic lesions | Palms and soles | NM | + | NM | Malignant melanoma on the base of the left thumb |  | Wide local resection with skin grafting for MM occurring in a keratotic lesion on her left palm. | Recurrence in the keratotic lesion adjacent to the skin graft after 3 years of follow-up. |
| 76 | Yin et al.^[28]^ | 2014 | China | F/25y | 8mo | Diffuse, reddish palmoplantar hyperkeratosis | Dorsum of the hands/feet | Waists, the Achilles tendons | NM | NM | Mild erythrokeratoderma on the elbows and knees |  |  |  |
| 77 | Yin et al.^[28]^ | 2014 | China | M/13y | Birth | Diffuse, reddish palmoplantar hyperkeratosis | Dorsum of the hands/feet | Waists, the Achilles tendons | + | NM | Tinea pedis |  |  |  |
| 78 | Yin et al.^[28]^ | 2014 | China | M/17y | 6mo | Diffuse, reddish palmoplantar hyperkeratosis | Dorsum of the hands/feet | Waists, the Achilles tendons | + | NM | Tinea pedis |  |  |  |
| 79 | Yin et al.^[28]^ | 2014 | China | F/24y | 6mo | Diffuse, reddish palmoplantar hyperkeratosis | Dorsum of the hands/feet | Waists, the Achilles tendons | + | NM | Tinea pedis |  |  |  |
| 80 | Yin et al.^[28]^ | 2014 | China | M/11y | 1y | Diffuse, reddish palmoplantar hyperkeratosis | Dorsum of the hands/feet | Waists, the Achilles tendons | + | NM | Tinea pedis |  |  |  |
| 81 | Yin et al.^[28]^ | 2014 | China | F/8y | 1y | Diffuse, reddish palmoplantar hyperkeratosis | Dorsum of the hands/feet | Waists, the Achilles tendons | NM | NM | Mild erythrokeratoderma affecting the elbows and knees, mild pruritus |  |  |  |
| 82 | Yin et al.^[28]^ | 2014 | China | M/30y | 4y | Diffuse, reddish palmoplantar hyperkeratosis | Dorsum of the hands/feet | Waists, the Achilles tendons | + | NM | Onychomycosis, tinea pedis |  |  |  |
| 83 | Mizuno et al.^[29]^ | 2014 | Japan | M/33y | 1y | Report confirmed NPPK | NM | Knees, elbows | + | NM | NM |  |  |  |
| 84 | Mizuno et al.^[29]^ | 2014 | Japan | F/18y | Birth | Report confirmed NPPK | NM | Knees, elbows | + | NM | NM |  |  |  |
| 85 | Mizuno et al.^[29]^ | 2014 | Japan | F/1y | 2mo | Report confirmed NPPK | NM | Knees | - | NM | NM |  |  |  |
| 86 | Mizuno et al.^[29]^ | 2014 | Japan | F/4y | Birth | Report confirmed NPPK | NM | Knees | - | NM | NM |  |  |  |
| 87 | Mizuno et al.^[29]^ | 2014 | Japan | F/6y | 3mo | Report confirmed NPPK | NM | - | + | NM | NM |  |  |  |
| 88 | Mizuno et al.^[29]^ | 2014 | Japan | F/17y | Birth | Report confirmed NPPK | NM | - | + | NM | NM |  |  |  |
| 89 | Mizuno et al.^[29]^ | 2014 | Japan | F/27y | Infancy | Report confirmed NPPK | NM | - | - | NM | NM |  |  |  |
| 90 | Mizuno et al.^[29]^ | 2014 | Japan | F/3y | 3mo | Report confirmed NPPK | NM | - | + | NM | NM |  |  |  |
| 91 | Mizuno et al.^[29]^ | 2014 | Japan | M/56y | 3y | Report confirmed NPPK | NM | Knees, elbows | + | NM | NM |  |  |  |
| 92 | Mizuno et al.^[29]^ | 2014 | Japan | M/73y | 3y | Report confirmed NPPK | NM | - | + | NM | NM |  |  |  |
| 93 | Mizuno et al.^[29]^ | 2014 | Japan | M/47y | 5y | Report confirmed NPPK | NM | Knees | - | NM | NM | P92' son |  |  |
| 94 | Mizuno et al.^[29]^ | 2014 | Japan | M/43y | 5y | Report confirmed NPPK | NM | - | + | NM | NM | P92' son |  |  |
| 95 | Mizuno et al.^[29]^ | 2014 | Japan | F/38y | 5y | Report confirmed NPPK | NM | Knees | - | NM | NM | P92' daughter |  |  |
| 96 | Park et al.^[30]^ | 2013 | Japan | F/9y | 3 y | Bilateral reddish, palmoplantar, hyperkeratotic lesions with erythematous borders | Extensor surface of the fingers and toes | Knees, elbows | + | NM | Distinct odor and maceration | Her sister |  |  |
| 97 | Park et al.^[30]^ | 2013 | Japan | F/6y | 3 y | Bilateral reddish, palmoplantar, hyperkeratotic lesions with erythematous borders | Extensor surface of the fingers and toes | NM | + | NM | NM | P96' sister |  |  |
| 98 | Kubo et al.^[31]^ | 2013 | Japan | F/10y | At birth | Report confirmed NPPK | NM | Knees | + | NM | NM |  |  |  |
| 99 | Kubo et al.^[31]^ | 2013 | Japan | F/2y | Early infancy | Report confirmed NPPK | NM | - | - | NM | NM |  |  |  |
| 100 | Kubo et al.^[31]^ | 2013 | Japan | M/31y | 1wk | Report confirmed NPPK | NM | Knees | + | NM | NM |  |  |  |
| 101 | Kubo et al.^[31]^ | 2013 | Japan | F/5y | Birth | Report confirmed NPPK | NM | Knees, elbows | + | NM | NM |  |  |  |
| 102 | Kubo et al.^[31]^ | 2013 | Japan | M/31y | Birth | Report confirmed NPPK | NM | Knees, elbows | + | NM | NM |  |  |  |
| 103 | Kubo et al.^[31]^ | 2013 | Japan | M/14y | 9-10y | Report confirmed NPPK | NM | Knees, elbows | + | NM | NM |  |  |  |
| 104 | Kubo et al.^[31]^ | 2013 | Japan | M/38y | Birth | Report confirmed NPPK | NM | Knees, elbows | + | NM | NM |  |  |  |
| 105 | Kubo et al.^[31]^ | 2013 | Japan | F/16y | Birth | Report confirmed NPPK | NM | - | + | NM | NM |  |  |  |
| 106 | Kubo et al.^[31]^ | 2013 | Japan | F/30y | Birth | Report confirmed NPPK | NM | Knees | + | NM | NM |  |  |  |
| 107 | Kubo et al.^[31]^ | 2013 | Japan | F/28y | 2y | Report confirmed NPPK | NM | - | + | NM | NM |  |  |  |
| 108 | Kubo et al.^[31]^ | 2013 | Japan | F/64y | Early infancy | Report confirmed NPPK | NM | - | - | NM | NM |  |  |  |
| 109 | Kubo et al.^[31]^ | 2013 | Japan | M/20y | Birth | Report confirmed NPPK | NM | Knees, elbows | + | NM | NM |  |  |  |
| 110 | Kubo et al.^[31]^ | 2013 | Japan | M/51y | 5–6y | Report confirmed NPPK | NM | Knees, elbows | + | NM | NM |  |  |  |
| 111 | Nakamizo et al.^[32]^ | 2012 | Japan | M/1y | 3mo | Bilateral, reddish, palmoplantar, hyperkeratotic lesions with an erythematous border | Palms and soles | NM | - | NM | Recurrent episodes of tinea pedis, toenail dystrophy |  |  |  |
| 112 | Nonomura et al.^[33]^ | 2012 | Japan | F/8y | Birth | Bilateral reddish, palmar and plantar hyperkeratotic lesions without itching or pain | Dorsum of the hands and feet, | Ears, knees, elbows, Achilles’ tendon | + | NM | Distinct odor and maceration |  | Tacrolimus ointment | Efficiently controlled |
| 113 | Sakabe et al.^[34]^ | 2009 | Japan | NM |  | Erythematous | Both hands | NM | NM | NM | NM |  | 0.1% tacrolimus ointment to the left hand and 0.05% betamethasone butyrate propionate ointment to the right hand (high-potency 0.12%betamethasone valerate ointment) | Both treatments, but improvement was greater on the left than on the right hand (Limited mild improvement) |
| 114 | Isoda et al.^[35]^ | 2009 | Japan | F/31y | First 3 years of their lives and gradually progressed until their late teens | Bilateral reddish | Palms and soles | NM | + | NM | Distinct odor, maceration, asthma, congenital ptosis |  |  |  |
| 115 | Isoda et al.^[35]^ | 2009 | Japan | F/29y | First 3 years of their lives and gradually progressed until their late teens | Bilateral reddish | Palms and soles | NM | + | NM | Distinct odor and maceration |  |  |  |
| 116 | Kabashima et al.^[36]^ | 2008 | Japan | M/17y | Infancy | Bilateral reddish, palmoplantar hyperkeratotic lesions | Dorsum of the hands and the Achilles tendon area | - | + | NM | Distinct odor, maceration, associated contact dermatitis | Isolated | Topical 0.1%betamethasone valerate ointment and keratolytic moisturizing creams |  |
| 117 | Minagawa et al.^[37]^ | 2005 | Japan | M/30y | Infancy | Report confirmed NPPK | NM | Knees, elbows | + | NM | NM | His brother |  |  |
| 118 | Chikenji et al.^[38]^ | 2005 | Japan | F/12y | Infancy | Report confirmed NPPK | NM | - | + | NM | NM | Her sister |  |  |
| 119 | Chikenji et al.^[38]^ | 2005 | Japan | F/7y | Infancy | Report confirmed NPPK | NM | - | + | NM | NM | P118' sister |  |  |
| 120 | Matsunaga et al.^[39]^ | 2000 | Japan | F/3y | Birth | Report confirmed NPPK | NM | Knees | + | NM | NM | Her sister |  |  |
| 121 | Matsunaga et al.^[39]^ | 2000 | Japan | F/10mo | Birth | Report confirmed NPPK | NM | - | - | NM | NM | P120' sister |  |  |
| 122 | Mitsuishi.^[40]^ | 1995 | Japan | M/6y | Birth | Report confirmed NPPK | NM | Knees, elbows | + | NM | NM | His brother |  |  |
| 123 | Mitsuishi.^[40]^ | 1995 | Japan | M/9y | Birth | Report confirmed NPPK | NM | Knees, elbows | + | NM | NM | P122' brother |  |  |
| 124 | Miyachi et al.^[41]^ | 1994 | Japan | F/11y | 4mo | Report confirmed NPPK | NM | Knees | + | NM | NM | Isolated case |  |  |
| 125 | Kitajima et al.^[42]^ | 1994 | Japan | M/6y | 3y | Report confirmed NPPK | NM | Knees, elbows | - | NM | NM | Isolated case |  |  |
| 126 | Kitajima et al.^[42]^ | 1994 | Japan | M/11mo | 2wk | Report confirmed NPPK | NM | Knee | NM | NM | NM | Isolated case |  |  |
| 127 | Kitajima et al.^[42]^ | 1994 | Japan | M/1y | 3mo | Report confirmed NPPK | NM | Knees, elbows | NM | NM | NM | His sister |  |  |
| 128 | Kitajima et al.^[42]^ | 1994 | Japan | F/5y | 1y | Report confirmed NPPK | NM | - | NM | NM | NM | P127' sister |  |  |
| 129 | Shimizu, N^[43]^ | 1992 | Japan | F/1y | 2mo | Report confirmed NPPK | NM | Knee | + | NM | NM | Her brother |  |  |
| 130 | Mitsuhashi et al.^[44]^ | 1989 | Japan | M/15y | 1mo | Report confirmed NPPK | NM | Knees | + | NM | NM | His sister |  |  |
| 131 | Mitsuhashi et al.^[44]^ | 1989 | Japan | F/13y | Birth | Report confirmed NPPK | NM | Knees, elbows | + | NM | NM | P130' sister |  |  |
| 132 | Aso K^[45]^ | 1989 | Japan | F/27y | Infancy | Report confirmed NPPK | NM | Knees, elbows | NM | NM | NM | Her brother |  |  |
| 133 | Nakamura et al.^[46]^ | 1981 | Japan | M/37y | Infancy | Report confirmed NPPK | NM | Knees, elbows | + | NM | NM | His sister |  |  |
| 134 | Nakamura et al.^[46]^ | 1981 | Japan | F/34y | Infancy | Report confirmed NPPK | NM | - | NM | NM | NM | P133' sister |  |  |

M, male; F, female; y, years; m, months; w, weeks; NM, not mentioned; +, present; −, not present; NPPK, Nagashima-type palmoplantar keratosis; P, patient.

**References**

1. Zhao, J., et al., *SERPINB7 novel mutation in Chinese patients with Nagashima-type palmoplantar keratosis and cases associated with atopic dermatitis.* International journal of dermatology, 2020.

2. Zhao, J., *Analysis of SERPINB7 Gene Mutation in Two Cases with Nagashima-type Palmoplantar Keratosis [in Chinese].* Inner Monglia Medical Journal, 2019. **051**(007): p. 784-785.

3. Sun, Z., et al., *Nagashima-type palmoplantar keratoderma:a case of homozygous deletion of the SERPINB7 gene mutation [in Chinese].* Journal of Dermatology and Venereology, 2019. **41**(02): p. 6-8.

4. Katayama, S., et al., *A Case of Malignant Melanoma Arising in Nagashima-type Palmoplantar Keratosis.* Acta dermato-venereologica, 2019. **99**(13): p. 1311-1312.

5. Hannula-Jouppi, K., et al., *Nagashima-type palmoplantar keratosis in Finland caused by a SERPINB7 founder mutation.* Journal of the American Academy of Dermatology, 2020. **83**(2): p. 643-645.

6. Korekawa, A., et al., *Nagashima-type palmoplantar keratoderma and malignant melanoma in Japanese patients.* The British journal of dermatology, 2019. **180**(2): p. 415-416.

7. Chassain, K., et al., *[Nagashima-type palmoplantar keratoderma: A little-known palmoplantar keratoderma in Europe].* Annales de dermatologie et de venereologie, 2019. **146**(2): p. 125-130.

8. Matsudate, Y., et al., *Coexistence of X-linked ichthyosis and Nagashima-type palmoplantar keratosis: A case report.* The Journal of dermatology, 2019. **46**(2): p. e54-e55.

9. Liu, C. and C. Li, *Analysis of SERPINB7 gene mutation in a pedigree of Nagashima-type palmoplantar keratosis [in Chinese].* Diagnosis and Therapy Journal of Dermato-Venereology, 2018. **025**(001): p. 8-11.

10. Hua, S., et al., *A novel frameshift SERPINB7 mutation in a Chinese case with Nagashima-type palmoplantar keratosis: case report and review of the literature.* Clinical experimental dermatology, 2018. **43**(8): p. 953-955.

11. Kogame, T., et al., *A follow-up report of acral melanoma in a patient with Nagashima-type palmoplantar keratosis: validation of SERPINB7 mutation and local recurrence.* European journal of dermatology : EJD, 2018. **28**(4): p. 519-520.

12. Ohguchi, Y., et al., *Gentamicin-Induced Readthrough and Nonsense-Mediated mRNA Decay of SERPINB7 Nonsense Mutant Transcripts.* The Journal of investigative dermatology, 2018. **138**(4): p. 836-843.

13. Yamauchi, A., et al., *Three cases of Nagashima-type palmoplantar keratosis associated with atopic dermatitis: A diagnostic pitfall.* The Journal of dermatology, 2018. **45**(5): p. e112-e113.

14. Yang, M., et al., *Analysis of SERPINB7 gene mutation in one case with Nagashima-type palmoplantar keratosis [in Chinese].* Chinese Youjiang Medical Journal, 2018. **46**(1): p. 23-25.

15. Dai, S., et al., *Nagashima-type Palmoplantar Keratoderma: Mutation Analysis of the SERPINB7 Gene [in Chinese].* CHINESE JOURNAL OF DERMATOVENEREOLOGY OF INTEGRATED TRADITIONAL AND WESTERN WESTERN MEDICINE, 2017(2).

16. Qiu, M. and X. Zou, *Progressive Nagashima-type palmoplantar keratosis in a Chinese patient with recurrent c.796C>T mutation in SERPINB7.* Indian journal of dermatology, venereology leprology, 2017. **83**(1): p. 136.

17. Tsutsumi, R., et al., *Nagashima-type palmoplantar keratosis with melanoma: absence of epidermal Langerhans cells in hyperkeratotic skin.* European journal of dermatology : EJD, 2017. **27**(2): p. 210-212.

18. Katsuno, M., et al., *Novel nonsense mutation in SERPINB7 and the treatment of foot odor in a patient with Nagashima-type palmoplantar keratosis.* The Journal of dermatology, 2017. **44**(7): p. e146-e147.

19. Hashimoto, T., et al., *Detection of SERPINB7 mutation can distinguish Nagashima-type palmoplantar keratoderma from other keratodermas with palmoplantar lesions.* Clinical and experimental dermatology, 2017. **42**(3): p. 342-345.

20. On, H., et al., *Identification of SERPINB7 mutations in Korean patients with Nagashima-type palmoplantar keratosis.* The Journal of dermatology, 2017. **44**(7): p. 840-841.

21. Zhang, J., et al., *Nagashima-type palmoplantar keratosis in a Chinese Han population.* Molecular medicine reports, 2016. **14**(5): p. 4049-4054.

22. Li, C., et al., *A sporadic case of Nagashima-type palmoplantar keratosis caused by gene mutation in SERPINB7.* Clinical and experimental dermatology, 2016. **41**(7): p. 811-3.

23. Nakajima, K., et al., *Novel frame-shift mutation in SERPINB7 in a Japanese patient with Nagashima-type palmoplantar keratosis.* The Journal of dermatology, 2017. **44**(7): p. 841-843.

24. Duo, L., et al., *Mutation analysis of the SERPINB7 gene in two patients with Nagashima-type palmoplantar keratoderma [in Chinese].* Chinese Journal of Dermatology, 2016. **49**(3): p. 180-182.

25. Miyauchi, T., et al., *Extensive Erythema and Hyperkeratosis on the Extremities and Lumbar Area as an Unusual Mani-festation of Nagashima-type Palmoplantar Keratosis.* Acta dermato-venereologica, 2016. **96**(6): p. 856-8.

26. Hida, T., et al., *Nagashima-type palmoplantar keratosis caused by compound heterozygous mutations in SERPINB7.* European journal of dermatology : EJD, 2015. **25**(2): p. 202-3.

27. Iwata, M., et al., *Malignant melanoma arising in the skin lesions of Nagashima-type palmoplantar keratosis.* European journal of dermatology : EJD, 2014. **24**(2): p. 259-60.

28. Yin, J., et al., *New and recurrent SERPINB7 mutations in seven Chinese patients with Nagashima-type palmoplantar keratosis.* The Journal of investigative dermatology, 2014. **134**(8): p. 2269-2272.

29. Mizuno, O., et al., *Highly prevalent SERPINB7 founder mutation causes pseudodominant inheritance pattern in Nagashima-type palmoplantar keratosis.* The British journal of dermatology, 2014. **171**(4): p. 847-53.

30. Park, K., K. Kabashima, and Y. Miyachi, *Possible nagashima-type palmoplantar keratosis in two siblings.* Case reports in dermatology, 2013. **5**(1): p. 58-60.

31. Kubo, A., et al., *Mutations in SERPINB7, encoding a member of the serine protease inhibitor superfamily, cause Nagashima-type palmoplantar keratosis.* American journal of human genetics, 2013. **93**(5): p. 945-56.

32. Nakamizo, S., et al., *Atypical nail dystrophy in a possible case of Nagashima-type palmoplantar keratosis.* The Journal of dermatology, 2012. **39**(5): p. 470-1.

33. Nonomura, Y., et al., *Suspected Nagashima-type palmoplantar keratosis with atypical hyperkeratotic lesions on the ears.* European journal of dermatology : EJD, 2012. **22**(3): p. 392-3.

34. Sakabe, J., et al., *Possible involvement of T lymphocytes in the pathogenesis of Nagashima-type keratosis palmoplantaris.* Clinical and experimental dermatology, 2009. **34**(7): p. e282-4.

35. Isoda, H., K. Kabashima, and Y. Tokura, *'Nagashima-type' keratosis palmoplantaris in two siblings.* Journal of the European Academy of Dermatology Venereology, 2009. **23**(6): p. 737-8.

36. Kabashima, K., et al., *"Nagashima-type" keratosis as a novel entity in the palmoplantar keratoderma category.* Archives of dermatology, 2008. **144**(3): p. 375-9.

37. Minagawa, Y., H. Saito, and A. Ishiko, *A case of keratosis palmoplantaris transgrediens Nagashima [in Japanese].* 59, 2005. **Hifuka No Rinsho**: p. 1168-1171.

38. Chikenji, T., et al., *Sibling cases of keratosis palmo-plantaris transgrediens Nagashima [in Japanese]. .* Proc Conference Disord Keratinization, 2005. **20**: p. 113-116.

39. Matsunaga, R. and M. Mizoguchi, *A sibling case of Nagashima-type of palmoplantar keratosis.* Rinsho Derma (Tokyo), 2000. **42**(13).

40. Mitsuishi, K., *A case of Nagashima-type keratosis palmoplantaris [in Japanese].* Proc Conference Disord Keratinization, 1995. **10**: p. 59-60.

41. Miyachi, M., et al., *Meleda type palmoplantar keratosis.* Hifubyoh Shinryou, 1994. **16**(12): p. 1121-1124.

42. Kitajima, S., S. Sugano, and T. Tsuji, *Meleda type of plamoplantar keratosis [in Japanese].* Proc Conference Disord Keratinization, 1994. **9**: p. 74-76.

43. Shimizu, N., *A case of “Meleda type” keratosis palmoplantaris [in Japanese].* Niigataken Ishikaiho, 1992. **7**(2): p. 2-10.

44. Mitsuhashi, Y., I. Hashimoto, and M. Takahashi, *Meleda type of keratosis palmoplantaris [in Japanese].* Hifubyoh Shinryou, 1989. **11**: p. 298-299.

45. Aso, K., *“Nagashima type” palmoplantar keratosis [in Japanese].* Hifuka Mook, 1989. **15**: p. 148-153.

46. Nakamura, M., et al., *A sibling case of Na-gashima-type of palmoplantar keratosis [in Japanese].* Rinsho Hifuka, 1981. **52**(6): p. 456-460.
