# Supplementary material for: Nagashima-Type Palmoplantar Keratosis: Clinical Characteristics, Genetic Characterization, and Clinical Management
Source: Biomed Res Int. 2021 Jan 27;2021:8841994. doi: 10.1155/2021/8841994 (PMC7861918; doi:10.1155/2021/8841994)
Supplement: Supplementary Materials — Supplement, Table 1: Clinical phenotypes in individuals with NPPK. Supplement, Table 2: SERPINB7 mutations in individuals with NPPK. [file 8841994.f1.zip › Supplement-Table 2 (1).docx]

**Supplement, Table 2:** SERPINB7 mutations in individuals with NPPK.

|  | | | | | | | | | **Molecular results** | | | |  |
| --- | --- | --- | --- | --- | --- | --- | --- | --- | --- | --- | --- | --- | --- |
|  |  |  |  |  |  |  |  |  | **Allele 1** | | **Allele 2** | |  |
| **No** | **Source** | **Year** | **Country** | **Sex/Age** | **Family history** | **Confirmed NPPK** | **Target gene** | **Genetic heterogeneity** | **Nucleotide change** | **Amino acid change** | **Nucleotide change** | **Amino acid change** | **Type of mutation** |
| 1 | Zhao et al.^[1]^ | 2020 | China | F/22mo |  | + | SERPINB7 | Compound heterozygous mutation | c.796C>T |  | c.521_522insT | Change the amino acid sequence starting with Val 175 and terminating the polypeptide 46 amino acids later. | Frame-shift mutation |
| 2 | Zhao et al.^[1]^ | 2020 | China | F | P1' sister | + | SERPINB7 | Compound heterozygous mutation | c.796C>T |  | c.521_522insT | Change the amino acid sequence starting with Val 175 and terminating the polypeptide 46 amino acids later. |  |
| 3 | Zhao et al.^[1]^ | 2020 | China | M/3y |  | + | SERPINB7 | Compound heterozygous mutation | c.796C>T |  | c.455G> T |  |  |
| 4 | Zhao et al.^[1]^ | 2020 | China | F/5y |  | + | SERPINB7 | Homozygous mutation | c.796C>T |  |  |  |  |
| 5 | Zhao et al.^[1]^ | 2020 | China | F |  | + | SERPINB7 | Homozygous nonsense mutation | c.796C>T |  |  |  |  |
| 6 | Zhao et al.^[1]^ | 2020 | China | M |  | + | SERPINB7 | Homozygous nonsense mutation | c.796C>T |  |  |  |  |
| 7 | Katayama et al.^[2]^ | 2019 | Japan  (Chinese P) | F/45y |  | + | SERPINB7 | Homozygous nonsense mutation | c.796C>T | p. Arg266Ter |  |  |  |
| 8 | Hannula-Jouppi et al.^[3]^ | 2019 | Finland | M/27y |  | + | SERPINB7 | Homozygous mutation | c.1136G>A | p. Cys379Tyr |  |  |  |
| 9 | Hannula-Jouppi et al.^[3]^ | 2019 | Finland | M/18y |  | + | SERPINB7 | Homozygous mutation | c.1136G>A | p. Cys379Tyr |  |  |  |
| 10 | Hannula-Jouppi et al.^[3]^ | 2019 | Finland | M/11y |  | + | SERPINB7 | Homozygous mutation | c.1136G>A | p. Cys379Tyr |  |  |  |
| 11 | Hannula-Jouppi et al.^[3]^ | 2019 | Finland | M/60y |  | + | SERPINB7 | Heterozygous carriers | c.1136G>A | p. Cys379Tyr |  |  |  |
| 12 | Hannula-Jouppi et al.^[3]^ | 2019 | Finland | F/21y |  | + | SERPINB7 | Heterozygous carriers | c.1136G>A | p. Cys379Tyr |  |  |  |
| 13 | Hannula-Jouppi et al.^[3]^ | 2019 | Finland | F/12y |  | + | SERPINB7 | Heterozygous carriers | c.1136G>A | p. Cys379Tyr |  |  |  |
| 14 | Hannula-Jouppi et al.^[3]^ | 2019 | Finland | M/16y |  | + | SERPINB7 | Heterozygous carriers | c.1136G>A | p. Cys379Tyr |  |  |  |
| 15 | Korekawa et al.^[4]^ | 2019 | Japan | F/65y |  | + | SERPINB7 | Compound heterozygous mutation | c.382C>T | p. Arg128Ter | c.455G>T | p. Gly152Val |  |
| 16 | Korekawa et al.^[4]^ | 2019 | Japan | F/85y |  | + | SERPINB7 | Homozygous nonsense mutation | c.796C>T | p. Arg266Ter |  |  |  |
| 17 | Korekawa et al.^[4]^ | 2019 | Japan | M/55y |  | + | SERPINB7 | Homozygous nonsense mutation | c.796C>T | p. Arg266Ter |  |  |  |
| 18 | Korekawa et al.^[4]^ | 2019 | Japan | M/38y |  | + | SERPINB7 | Compound heterozygous mutation | c.796C>T | p. Arg266Ter | c.830C>T | p. Pro277Leu |  |
| 19 | Chassain et al.^[5]^ | 2019 | France (Chinese P) | F/6y |  | + | SERPINB7 | Compound heterozygous mutation | c.650-653delCTGT | p. Ser217Leufs*7 | c.796C>T | p.R266* |  |
| 20 | Sun et al.^[6]^ | 2019 | China | F/29y | Consanguineous mating | + | SERPINB7 | Homozygous mutation | c.650-653delCTGT | p. S217Lfs*7 |  |  | Frame-shift mutation |
| 21 | Sun et al.^[6]^ | 2019 | China | F | P20' mother | - | SERPINB7 | Compound heterozygous mutation | c.650-653delCTGT | p. S217Lfs*7 |  |  |  |
| 22 | Sun et al.^[6]^ | 2019 | China | M | P20' father | - | SERPINB7 | Compound heterozygous mutation | c.650-653delCTGT | p. S217Lfs*7 |  |  |  |
| 23 | Zhao J^[7]^ | 2019 | China | M/4y, 8mo |  | + | SERPINB7 | Homozygous mutation | c.796C>T | p. Arg266Ter |  |  |  |
| 24 | Zhao J^[7]^ | 2019 | China | F | P23' mother | - | SERPINB7 | Heterozygous carriers | c.796C>T |  |  |  |  |
| 25 | Zhao J^[7]^ | 2019 | China | M/17y |  | + | SERPINB7 | Compound heterozygous mutation | c.796C>T |  | c.455G>T | p. Gly152Valfs* |  |
| 26 | Zhao J^[7]^ | 2019 | China | F | P25' mother | - | SERPINB7 | Heterozygous carriers | c.796C>T |  |  |  |  |
| 27 | Matsudate et al.^[8]^ | 2019 | Japan | M/9y |  | + | SERPINB7 | Heterozygous nonsense mutation | c.796C>T | p. Arg266Ter |  |  |  |
| 28 | Hua et al.^[9]^ | 2018 | China | M/6y |  | + | SERPINB7 | Compound heterozygous mutation | c.271delC | p.H91Tfs*9 |  |  | Frame-shift mutation |
| 29 | Kogame et al.^[10]^ | 2018 | Japan | F/64y |  | + | SERPINB7 | Compound heterozygous mutation | C.796C>T | p.R266* | C.218_219del2ins12 |  | A nonsense mutation (former)；a deletion/insertion mutation (latter) |
| 30 | Liu C, Li CX^[11]^ | 2018 | China | M/23y |  | + | SERPINB7 | Homozygous mutation | c.796C>T | p. Arg266Ter |  |  |  |
| 31 | Liu C, Li CX^[11]^ | 2018 | China | F | P30' mother | - | SERPINB7 | Homozygous mutation | c.796C>T | p. Arg266Ter |  |  |  |
| 32 | Ohguchi et al.^[12]^ | 2018 | Japan | M/28y |  | + | SERPINB7 | Homozygous mutation | c.796C>T | c.796C>T | c.830C>T | p. P277L |  |
| 33 | Ohguchi et al.^[12]^ | 2018 | Japan | M/29y |  | + | SERPINB7 | Homozygous mutation | c.796C>T | c.796C>T |  |  |  |
| 34 | Ohguchi et al.^[12]^ | 2018 | Japan | F/6y |  | + | SERPINB7 | Compound heterozygous mutation | c.796C>T | c.455-1G>A |  |  |  |
| 35 | Ohguchi et al.^[12]^ | 2018 | Japan | F/40y |  | + | SERPINB7 | Compound heterozygous mutation | c.796C>T | c.455-1G>A |  |  |  |
| 36 | Ohguchi et al.^[12]^ | 2018 | Japan | M/46y |  | + | SERPINB7 | Homozygous mutation | c.796C>T | c.796C>T |  |  |  |
| 37 | Qiu M, Zou XB^[13]^ | 2017 | China | F/28y |  | + | SERPINB7 | Homozygous mutation | c.796C>T | p. Arg266* |  |  |  |
| 38 | Qiu M, Zou XB^[13]^ | 2017 | China | F |  | + | SERPINB7 | Heterozygous carriers |  |  |  |  |  |
| 39 | Yamauchi et al.^[14]^ | 2017 | Japan | F/12y |  | + | SERPINB7 | Compound heterozygous mutation | c.796C>T | p.R266* | c.218_219del2ins12 | p. Gln73Leufs*17 |  |
| 40 | Yamauchi et al.^[14]^ | 2017 | Japan | M/20y |  | + | SERPINB7 | Compound heterozygous mutation | c.796C>T | p.R266* |  |  |  |
| 41 | Tsutsumi et al.^[15]^ | 2017 | Japan | F/63y |  | + | SERPINB7 | A nonsense mutation inexon8 | c.796C>T |  |  |  | A nonsense mutation |
| 42 | Katsuno et al.^[16]^ | 2017 | Japan | M/63y |  | + | SERPINB7 | Compound heterozygous mutations | c.218-219del2ins12 | p. Gln73Leufs*17 | c.382C>T | p.R128* | A nonsense mutation |
| 43 | Hashimoto et al.^[17]^ | 2017 | Japan |  |  | + | SERPINB7 | Homozygous mutation | c.796C>T | p. Arg266Ter |  |  |  |
| 44 | Hashimoto et al.^[17]^ | 2017 | Japan |  |  | + | SERPINB7 | Homozygous mutation | c.796C>T | p. Arg266Ter |  |  |  |
| 45 | Hashimoto et al.^[17]^ | 2017 | Japan |  |  | + | SERPINB7 | Homozygous mutation | c.218_219del2ins12 | p. Gln73LeufsTer17 |  |  |  |
| 46 | Hashimoto et al.^[17]^ | 2017 | Japan |  |  | + | SERPINB7 | Compound heterozygous mutation | c.218_219del2ins12 | p. Gln73LeufsTer17 | c.796C>T | p. Arg266Ter |  |
| 47 | Hashimoto et al.^[17]^ | 2017 | Japan |  |  | + | SERPINB7 | Compound heterozygous mutation | c.455G>A | p. Gly152ValfsTer21 | c.796C>T | p. Arg266Ter |  |
| 48 | Hashimoto et al.^[17]^ | 2017 | Japan |  |  | + | SERPINB7 | Compound heterozygous mutation | c.796C>T | p. Arg266Ter | c.830C>T | p. Pro277Leu |  |
| 49 | Hashimoto et al.^[17]^ | 2017 | Japan |  |  | + | SERPINB7 | Compound heterozygous mutation | c.796C>T | p. Arg266Ter | c.830C>T | p. Pro277Leu |  |
| 50 | Hashimoto et al.^[17]^ | 2017 | Japan |  |  | Undetermined | SERPINB7 | Compound heterozygous mutation | c.796C>T | p. Arg266Ter | c.830C>T | p. Pro277Leu |  |
| 51 | Hashimoto et al.^[17]^ | 2017 | Japan |  |  | + | SERPINB7 | Compound heterozygous mutation | c.796C>T | p. Arg266Ter |  |  |  |
| 52 | Hashimoto et al.^[17]^ | 2017 | Japan |  |  | Undetermined | SERPINB7 | Compound heterozygous mutation | c.796C>T | p. Arg266Ter |  |  |  |
| 53 | On et al.^[18]^ | 2017 | Korean | M |  | + | SERPINB7 | Compound heterozygous mutation | c.522-523insT | p. Val175Cysfs*46 | c.796C>T | p. Arg266Ter | Frame-shift mutation |
| 54 | On et al.^[18]^ | 2017 | Korean | M |  | + | SERPINB7 | Homozygous mutation | c.796C>T |  |  |  | Recurrent pathogenic mutation |
| 55 | On et al.^[18]^ | 2017 | Korean | F |  | + | SERPINB7 | Homozygous mutation | c.796C>T |  |  |  | Recurrent nonsense mutation |
| 56 | Yang et al.^[19]^ | 2017 | China | M/18y |  | + | SERPINB7 | Compound heterozygous mutation | c.796C>T | p. Arg266Ter |  |  |  |
| 57 | Yang et al.^[19]^ | 2017 | China | F | P56' mother | - | SERPINB7 | Compound heterozygous mutation | c.796C>T | p. Arg266Ter |  |  |  |
| 58 | Yang et al.^[19]^ | 2017 | China | M | P56' father | - | SERPINB7 | Compound heterozygous mutation | c.796C>T | p. Arg266Ter |  |  |  |
| 59 | Dai et al.^[20]^ | 2017 | China | M/17y |  | + | SERPINB7 | Compound heterozygous mutation | c.796C>T | p. R266* | c.455G>T |  |  |
| 60 | Dai et al.^[20]^ | 2017 | China | F | P59' mother | - | SERPINB7 | Compound heterozygous mutation |  |  | c.455G>T |  |  |
| 61 | Dai et al.^[20]^ | 2017 | China | M/26y |  | + | SERPINB7 | Compound heterozygous mutation | c.796C>T | p.R266* | c.455G>T |  |  |
| 62 | Zhang et al.^[21]^ | 2016 | China | M/8m |  | + | SERPINB7 | Homozygous founder mutations | c.796C>T | p.R266* | c.796C>T | p.R266* |  |
| 63 | Zhang et al.^[21]^ | 2016 | China | F/2y |  | + | SERPINB7 | Compound heterozygous mutations | c.796C>T | p.R266* | c.522dupT | p. Val175fs |  |
| 64 | Zhang et al.^[21]^ | 2016 | China | F/19y |  | + | SERPINB7 | Homozygous founder mutations | c.796C>T | p.R266* | c.796C>T | p.R266* |  |
| 65 | Zhang et al.^[21]^ | 2016 | China | M/4y |  | + | SERPINB7 | Homozygous founder mutations | c.796C>T | p.R266* | c.796C>T | p.R266* |  |
| 66 | Zhang et al.^[21]^ | 2016 | China | M/16y |  | + | SERPINB7 | One heterozygous founder mutation | c.796C>T | p.R266* |  |  |  |
| 67 | Zhang et al.^[21]^ | 2016 | China | F/26y |  | + | SERPINB7 | Homozygous founder mutations | c.796C>T | p.R266* | c.796C>T | p.R266* |  |
| 68 | Zhang et al.^[21]^ | 2016 | China | F/17y |  | + | SERPINB7 | One heterozygous founder mutation | c.796C>T | p.R266* |  |  |  |
| 69 | Zhang et al.^[21]^ | 2016 | China | F/24y |  | + | SERPINB7 | One heterozygous founder mutation | c.796C>T | p.R266* |  |  |  |
| 70 | Zhang et al.^[21]^ | 2016 | China | F/36y |  | + | SERPINB7 | Homozygous founder mutations | c.796C>T | p.R266* | c.796C>T | p.R266* |  |
| 71 | Zhang et al.^[21]^ | 2016 | China | M/22y |  | + | SERPINB7 | Compound heterozygous mutation | c.796C>T | p.R266* | c.122_127delTGGTCC | p. Leu41fs | In-frame deletion mutation |
| 72 | Zhang et al.^[21]^ | 2016 | China | F/51y |  | + | SERPINB7 | Compound heterozygous mutation | c.796C>T | p.R266* | c.522dupT | p. Val175fs |  |
| 73 | Zhang et al.^[21]^ | 2016 | China | F/2y |  | + | SERPINB7 | Compound heterozygous mutation | c.796C>T | p.R266* | c.455G>T | Predicted splicing alteration |  |
| 74 | Li et al.^[22]^ | 2016 | China | M/45y |  | + | SERPINB7 | Homozygous nonsense mutation | c.796C>T | p. Arg266Ter |  |  |  |
| 75 | Nakajima et al.^[23]^ | 2016 | Japan | M/12y |  | + | SERPINB7 | Compound heterozygous mutation | c.796C>T | p.R266 * | c.635delG | p.K213Sfs*12 | Frame-shift mutation |
| 76 | Miyauchi et al.^[24]^ | 2016 | Japan | M/18y |  | + | SERPINB7 | Homozygous nonsense mutation | c.796C>T | p. Arg266Ter |  |  |  |
| 77 | Miyauchi et al.^[24]^ | 2016 | Japan | M | P76' father | Undetermined | SERPINB7 | Homozygous nonsense mutation | c.796C>T | p. Arg266Ter | c.797G>A | Non-pathogenic single-nucleotide polymorphism (SNP) |  |
| 78 | Miyauchi et al.^[24]^ | 2016 | Japan | F | P76' mother | Undetermined | SERPINB7 | Compound heterozygous mutation | c.796C>T | p. Arg266Ter |  |  |  |
| 79 | Duo et al.^[25]^ | 2016 | China | F/24y |  | + | SERPINB7 | Homozygous mutation | c.796C>T | p.R266* |  |  |  |
| 80 | Duo et al.^[25]^ | 2016 | China | F | P79' mother | - | SERPINB7 | Heterozygous carriers | c.796C>T |  |  |  |  |
| 81 | Duo et al.^[25]^ | 2016 | China | M | P79' father | - | SERPINB7 | Heterozygous carriers | c.796C>T |  |  |  |  |
| 82 | Duo et al.^[25]^ | 2016 | China | F/25y |  | + | SERPINB7 | Homozygous mutation | c.796C>T | p.R266* |  |  |  |
| 83 | Duo et al.^[25]^ | 2016 | China | F | P82' mother | - | SERPINB7 | Heterozygous carriers | c.796C>T |  |  |  |  |
| 84 | Duo et al.^[25]^ | 2016 | China | M | P82' father | - | SERPINB7 | Heterozygous carriers | c.796C>T |  |  |  |  |
| 85 | Shiohama et al.^[26]^ | 2016 | Japan | F |  | + | SERPINB7 | Compound heterozygous mutation | c.796C>T | p.R266* | c.830C>T | p. P277L | Missense mutation |
| 86 | Shiohama et al.^[26]^ | 2016 | Japan | M |  | + | SERPINB7 | Compound heterozygous mutation | c.796C>T | p.R266* | c.830C>T | p. P277L | Recurrent mutations |
| 87 | Shiohama et al.^[26, 27]^ | 2016 | Japan | F |  | + | SERPINB7 | Compound heterozygous mutation | c.796C>T | p.R266* | c.830C>T | p. P277L |  |
| 88 | Shiohama et al.^[26, 27]^ | 2016 | Japan | F |  | + | SERPINB7 | Compound heterozygous mutation | c.218_219delAGinsTAAACTTTACCT |  | c.830C>T | p. P277L |  |
| 89 | Shiohama et al.^[26, 27]^ | 2016 | Japan | F |  | + | SERPINB7 | Compound heterozygous mutation | c.218_219delAGinsTAAACTTTACCT |  | c.830C>T | p. P277L |  |
| 90 | Shiohama et al.^[26, 28]^ | 2016 | Japan | M |  | + | SERPINB7 | Compound heterozygous mutation | c.455-1G>A |  | c.830C>T | p. P277L |  |
| 91 | Hida et al.^[29]^ | 2015 | Japan | F/16y |  | + | SERPINB7 | Compound heterozygous mutation | c.455-1G>A |  | c.455G>T |  |  |
| 92 | Yin et al.^[30]^ | 2014 | China | F/25y |  | + | SERPINB7 | Homozygous mutation | c.796C>T | p.R266^*^ | c.796C>T | p.R266^*^ |  |
| 93 | Yin et al.^[30]^ | 2014 | China | F | P92' father | - |  | Heterozygous carriers |  |  |  |  |  |
| 94 | Yin et al.^[30]^ | 2014 | China | M | P92' mother | - |  | Heterozygous carriers |  |  |  |  |  |
| 95 | Yin et al.^[30]^ | 2014 | China | M/13y |  | + | SERPINB7 | Homozygous mutation | c.796C>T | p.R266^*^ | c.796C>T | p.R266^*^ |  |
| 96 | Yin et al.^[30]^ | 2014 | China | F | P95' father | - |  | Heterozygous carriers |  |  |  |  |  |
| 97 | Yin et al.^[30]^ | 2014 | China | M | P95' mother | - |  | Heterozygous carriers |  |  |  |  |  |
| 98 | Yin et al.^[30]^ | 2014 | China | M/17y |  | + | SERPINB7 | Homozygous mutation | c.796C>T | p.R266^*^ | c.796C>T | p.R266^*^ |  |
| 99 | Yin et al.^[30]^ | 2014 | China | F | P98' father | - |  | Heterozygous carriers |  |  |  |  |  |
| 100 | Yin et al.^[30]^ | 2014 | China | M | P98' mother | - |  | Heterozygous carriers |  |  |  |  |  |
| 101 | Yin et al.^[30]^ | 2014 | China | F/24y |  | + | SERPINB7 | Homozygous mutation | c.796C>T | p.R266^*^ | c.796C>T | p.R266^*^ |  |
| 102 | Yin et al.^[30]^ | 2014 | China | F | P101' father | - |  | Heterozygous carriers |  |  |  |  |  |
| 103 | Yin et al.^[30]^ | 2014 | China | M | P101' mother | - |  | Heterozygous carriers |  |  |  |  |  |
| 104 | Yin et al.^[30]^ | 2014 | China | M/11y |  | + | SERPINB7 | Compound heterozygous mutation | c.796C>T | p.R266^*^ | c.650-653delCTGT | p. S217Lfs*7 | Frame-shift mutation |
| 105 | Yin et al.^[30]^ | 2014 | China | F/8y |  | + | SERPINB7 | Compound heterozygous mutation | c.796C>T | p.R266^*^ | c.455G>T | p. G152V | Point mutation |
| 106 | Yin et al.^[30]^ | 2014 | China | F | P105' father | - |  | Heterozygous carriers |  |  |  |  |  |
| 107 | Yin et al.^[30]^ | 2014 | China | M | P105' mother | - |  | Heterozygous carriers |  |  |  |  |  |
| 108 | Yin et al.^[30]^ | 2014 | China | M/30y |  | + | SERPINB7 | Homozygous mutation | c.522-523insT | p. V175Cfs^*^46 | c.522-523insT | Splicing alternation | Frame-shift mutation |
| 109 | Yin et al.^[30]^ | 2014 | China | F | P108' father | - |  | Heterozygous carriers |  |  |  |  |  |
| 110 | Yin et al.^[30]^ | 2014 | China | M | P108' mother | - |  | Heterozygous carriers |  |  |  |  |  |
| 111 | Mizuno et al.^[31]^ | 2014 | Japan | M/33y |  | + | SERPINB7 | Homozygous mutation | c.796C>T | p. Arg266Ter | c.796C>T | p. Arg266Ter |  |
| 112 | Mizuno et al.^[31]^ | 2014 | Japan | F/18y |  | + | SERPINB7 | Homozygous mutation | c.796C>T | p. Arg266Ter | c.796C>T | p. Arg266Ter |  |
| 113 | Mizuno et al.^[31]^ | 2014 | Japan | F/1y |  | + | SERPINB7 | Homozygous mutation | c.796C>T | p. Arg266Ter | c.796C>T | p. Arg266Ter |  |
| 114 | Mizuno et al.^[31]^ | 2014 | Japan | F/4y |  | + | SERPINB7 | Compound heterozygous mutation | c.796C>T | p. Arg266Ter | c.218_219delinsTAAACTTTACCT | p.? |  |
| 115 | Mizuno et al.^[31]^ | 2014 | Japan | F/6y |  | + | SERPINB7 | Compound heterozygous mutation | c.796C>T | p. Arg266Ter | c.218_219delinsTAAACTTTACCT | p.? |  |
| 116 | Mizuno et al.^[31, 32]^ | 2014 | Japan | F/17y |  | + | SERPINB7 | Compound heterozygous mutation | c.796C>T | p. Arg266Ter | c.218_219delinsTAAACTTTACCT | p.? |  |
| 117 | Mizuno et al.^[31]^ | 2014 | Japan | F/27y |  | + | SERPINB7 | Compound heterozygous mutation | c.796C>T | p. Arg266Ter | c.455-1G>A | p.? |  |
| 118 | Mizuno et al.^[31, 32]^ | 2014 | Japan | F/3y |  | + | SERPINB7 | Compound heterozygous mutation | c.796C>T | p. Arg266Ter | c.455-1G>A | p.? |  |
| 119 | Mizuno et al.^[31]^ | 2014 | Japan | M/56y |  | + | SERPINB7 | Compound heterozygous mutation | c.796C>T | p. Arg266Ter | c.336+2T>G | p.? | Splice donor mutation |
| 120 | Mizuno et al.^[31]^ | 2014 | Japan | M/73y |  | + | SERPINB7 | Homozygous mutation | c.796C>T | p. Arg266Ter | c.796C>T | p. Arg266Ter |  |
| 121 | Mizuno et al.^[31]^ | 2014 | Japan | M/47y |  | + | SERPINB7 | Homozygous mutation | c.796C>T | p. Arg266Ter | c.796C>T | p. Arg266Ter |  |
| 122 | Mizuno et al.^[31]^ | 2014 | Japan | M/43y |  | + | SERPINB7 | Homozygous mutation | c.796C>T | p. Arg266Ter | c.796C>T | p. Arg266Ter |  |
| 123 | Mizuno et al.^[31]^ | 2014 | Japan | F/38y |  | + | SERPINB7 | Homozygous mutation | c.796C>T | p. Arg266Ter | c.796C>T | p. Arg266Ter |  |
| 124 | Kubo et al.^[33]^ | 2013 | Japan | F/10y |  | + | SERPINB7 | Homozygous mutation | c.796C>T | p. Arg266* | c.796C>T | p. Arg266* | A nonsense mutation |
| 125 | Kubo et al.^[33]^ | 2013 | Japan | F/2y |  | + | SERPINB7 | Homozygous mutation | c.796C>T | p. Arg266* | c.796C>T | p. Arg266* |  |
| 126 | Kubo et al.^[33]^ | 2013 | Japan | M/31y |  | + | SERPINB7 | Homozygous mutation | c.796C>T | p. Arg266* | c.796C>T | p. Arg266* |  |
| 127 | Kubo et al.^[33]^ | 2013 | Japan | F/5y |  | + | SERPINB7 | Homozygous mutation | c.796C>T | p. Arg266* | c.796C>T | p. Arg266* |  |
| 128 | Kubo et al.^[33]^ | 2013 | Japan | M/31y |  | + | SERPINB7 | Homozygous mutation | c.796C>T | p. Arg266* | c.796C>T | p. Arg266* |  |
| 129 | Kubo et al.^[33]^ | 2013 | Japan | M/14y |  | + | SERPINB7 | Homozygous mutation | c.796C>T | p. Arg266* | c.796C>T | p. Arg266* |  |
| 130 | Kubo et al.^[33]^ | 2013 | Japan | M/38y |  | + | SERPINB7 | Compound heterozygous mutation | c.796C>T | p. Arg266* | c.218_219delAGinsTAAACTTTACCT (c.218_219del2ins12) | p. Gln73Leufs*17b | Small indel mutation |
| 131 | Kubo et al.^[33]^ | 2013 | Japan | F/16y |  | + | SERPINB7 | Compound heterozygous mutation | c.796C>T | p. Arg266* | c.218_219delAGinsTAAACTTTACCT (c.218_219del2ins12) | p. Gln73Leufs*17b |  |
| 132 | Kubo et al.^[33]^ | 2013 | Japan | F/30y |  | + | SERPINB7 | Compound heterozygous mutation | c.796C>T | p. Arg266* | c.218_219delAGinsTAAACTTTACCT (c.218_219del2ins12) | p. Gln73Leufs*17b |  |
| 133 | Kubo et al.^[33]^ | 2013 | Japan | F/28y |  | + | SERPINB7 | Compound heterozygous mutation | c.796C>T | p. Arg266* | c.218_219delAGinsTAAACTTTACCT (c.218_219del2ins12) | p. Gln73Leufs*17b |  |
| 134 | Kubo et al.^[33]^ | 2013 | Japan | F/64y |  | + | SERPINB7 | Compound heterozygous mutation | c.796C>T | p. Arg266* | c.218_219delAGinsTAAACTTTACCT (c.218_219del2ins12) | p. Gln73Leufs*17b |  |
| 135 | Kubo et al.^[33]^ | 2013 | Japan | M/20y |  | + | SERPINB7 | Compound heterozygous mutation | c.796C>T | p. Arg266* | c.455-1G>A | p. Gly152Valfs*21b | Splice acceptor site |
| 136 | Kubo et al.^[33]^ | 2013 | Japan | M/51y |  | + | SERPINB7 | Compound heterozygous mutation | c.796C>T | p. Arg266* | c.455-1G>A | p. Gly152Valfs*21b | Splice acceptor site |
| 137 | Kubo et al.^[33]^ | 2013 | Non-Asian populations |  |  | - | SERPINB7 | NM | c.309delT | p. Phe103Leufs*33 |  |  |  |
| 138 | Kubo et al.^[33]^ | 2013 | China |  |  | - | SERPINB7 | Compound heterozygous mutation | c.336+2T>G |  |  |  |  |

SERPINB7, serpin peptidase inhibitor 7; NPPK, Nagashima-type palmoplantar keratosis; M, male; F, female; y, years; m, months; w, weeks; NM, not mentioned; +, present; −, not present; NPPK, Nagashima-type palmoplantar keratosis; P, patient.

**References**

1. Zhao, J., et al., *SERPINB7 novel mutation in Chinese patients with Nagashima-type palmoplantar keratosis and cases associated with atopic dermatitis.* International journal of dermatology, 2020.

2. Katayama, S., et al., *A Case of Malignant Melanoma Arising in Nagashima-type Palmoplantar Keratosis.* Acta dermato-venereologica, 2019. **99**(13): p. 1311-1312.

3. Hannula-Jouppi, K., et al., *Nagashima-type palmoplantar keratosis in Finland caused by a SERPINB7 founder mutation.* Journal of the American Academy of Dermatology, 2020. **83**(2): p. 643-645.

4. Korekawa, A., et al., *Nagashima-type palmoplantar keratoderma and malignant melanoma in Japanese patients.* The British journal of dermatology, 2019. **180**(2): p. 415-416.

5. Chassain, K., et al., *[Nagashima-type palmoplantar keratoderma: A little-known palmoplantar keratoderma in Europe].* Annales de dermatologie et de venereologie, 2019. **146**(2): p. 125-130.

6. Sun, Z., et al., *Nagashima-type palmoplantar keratoderma:a case of homozygous deletion of the SERPINB7 gene mutation [in Chinese].* Journal of Dermatology and Venereology, 2019. **41**(02): p. 6-8.

7. Zhao, J., *Analysis of SERPINB7 Gene Mutation in Two Cases with Nagashima-type Palmoplantar Keratosis [in Chinese].* Inner Monglia Medical Journal, 2019. **051**(007): p. 784-785.

8. Matsudate, Y., et al., *Coexistence of X-linked ichthyosis and Nagashima-type palmoplantar keratosis: A case report.* The Journal of dermatology, 2019. **46**(2): p. e54-e55.

9. Hua, S., et al., *A novel frameshift SERPINB7 mutation in a Chinese case with Nagashima-type palmoplantar keratosis: case report and review of the literature.* Clinical experimental dermatology, 2018. **43**(8): p. 953-955.

10. Kogame, T., et al., *A follow-up report of acral melanoma in a patient with Nagashima-type palmoplantar keratosis: validation of SERPINB7 mutation and local recurrence.* European journal of dermatology : EJD, 2018. **28**(4): p. 519-520.

11. Liu, C. and C. Li, *Analysis of SERPINB7 gene mutation in a pedigree of Nagashima-type palmoplantar keratosis [in Chinese].* Diagnosis and Therapy Journal of Dermato-Venereology, 2018. **025**(001): p. 8-11.

12. Ohguchi, Y., et al., *Gentamicin-Induced Readthrough and Nonsense-Mediated mRNA Decay of SERPINB7 Nonsense Mutant Transcripts.* The Journal of investigative dermatology, 2018. **138**(4): p. 836-843.

13. Qiu, M. and X. Zou, *Progressive Nagashima-type palmoplantar keratosis in a Chinese patient with recurrent c.796C>T mutation in SERPINB7.* Indian journal of dermatology, venereology leprology, 2017. **83**(1): p. 136.

14. Yamauchi, A., et al., *Three cases of Nagashima-type palmoplantar keratosis associated with atopic dermatitis: A diagnostic pitfall.* 2018. **45**(5): p. e112-e113.

15. Tsutsumi, R., et al., *Nagashima-type palmoplantar keratosis with melanoma: absence of epidermal Langerhans cells in hyperkeratotic skin.* European journal of dermatology : EJD, 2017. **27**(2): p. 210-212.

16. Katsuno, M., et al., *Novel nonsense mutation in SERPINB7 and the treatment of foot odor in a patient with Nagashima-type palmoplantar keratosis.* The Journal of dermatology, 2017. **44**(7): p. e146-e147.

17. Hashimoto, T., et al., *Detection of SERPINB7 mutation can distinguish Nagashima-type palmoplantar keratoderma from other keratodermas with palmoplantar lesions.* Clinical and experimental dermatology, 2017. **42**(3): p. 342-345.

18. On, H., et al., *Identification of SERPINB7 mutations in Korean patients with Nagashima-type palmoplantar keratosis.* The Journal of dermatology, 2017. **44**(7): p. 840-841.

19. Yang, M., et al., *Analysis of SERPINB7 gene mutation in one case with Nagashima-type palmoplantar keratosis [in Chinese].* Chinese Youjiang Medical Journal, 2018. **46**(1): p. 23-25.

20. Dai, S., et al., *Nagashima-type Palmoplantar Keratoderma: Mutation Analysis of the SERPINB7 Gene [in Chinese].* CHINESE JOURNAL OF DERMATOVENEREOLOGY OF INTEGRATED TRADITIONAL AND WESTERN WESTERN MEDICINE, 2017(2).

21. Zhang, J., et al., *Nagashima-type palmoplantar keratosis in a Chinese Han population.* Molecular medicine reports, 2016. **14**(5): p. 4049-4054.

22. Li, C., et al., *A sporadic case of Nagashima-type palmoplantar keratosis caused by gene mutation in SERPINB7.* Clinical and experimental dermatology, 2016. **41**(7): p. 811-3.

23. Nakajima, K., et al., *Novel frame-shift mutation in SERPINB7 in a Japanese patient with Nagashima-type palmoplantar keratosis.* The Journal of dermatology, 2017. **44**(7): p. 841-843.

24. Miyauchi, T., et al., *Extensive Erythema and Hyperkeratosis on the Extremities and Lumbar Area as an Unusual Mani-festation of Nagashima-type Palmoplantar Keratosis.* Acta dermato-venereologica, 2016. **96**(6): p. 856-8.

25. Duo, L., et al., *Mutation analysis of the SERPINB7 gene in two patients with Nagashima-type palmoplantar keratoderma [in Chinese].* Chinese Journal of Dermatology, 2016. **49**(3): p. 180-182.

26. Shiohama, A., et al., *Identification and Characterization of a Recessive Missense Mutation p.P277L in SERPINB7 in Nagashima-Type Palmoplantar Keratosis.* The Journal of investigative dermatology, 2016. **136**(1): p. 325-8.

27. Isoda, H., K. Kabashima, and Y. Tokura, *'Nagashima-type' keratosis palmoplantaris in two siblings.* Journal of the European Academy of Dermatology Venereology, 2009. **23**(6): p. 737-8.

28. Kabashima, K., et al., *"Nagashima-type" keratosis as a novel entity in the palmoplantar keratoderma category.* Archives of dermatology, 2008. **144**(3): p. 375-9.

29. Hida, T., et al., *Nagashima-type palmoplantar keratosis caused by compound heterozygous mutations in SERPINB7.* European journal of dermatology : EJD, 2015. **25**(2): p. 202-3.

30. Yin, J., et al., *New and recurrent SERPINB7 mutations in seven Chinese patients with Nagashima-type palmoplantar keratosis.* The Journal of investigative dermatology, 2014. **134**(8): p. 2269-2272.

31. Mizuno, O., et al., *Highly prevalent SERPINB7 founder mutation causes pseudodominant inheritance pattern in Nagashima-type palmoplantar keratosis.* The British journal of dermatology, 2014. **171**(4): p. 847-53.

32. Suzuki, S., et al., *Identification of previously unknown SERPINB7 splice variants in patients with Nagashima-type palmoplantar keratosis reveals the importance of the CD-loop of SERPINB7.* The British journal of dermatology, 2015. **173**(5): p. 1288-90.

33. Kubo, A., et al., *Mutations in SERPINB7, encoding a member of the serine protease inhibitor superfamily, cause Nagashima-type palmoplantar keratosis.* American journal of human genetics, 2013. **93**(5): p. 945-56.
